# Taxonomic and Functional Profiles of the Gastrointestinal Microbiome Across Digestive Tract Segments in Growing Water Buffaloes

**DOI:** 10.3390/microorganisms14102206

**Published:** 2026-10-01

**Authors:** Jingjing Wen, Jiajie Huang, Guixian Liu, Shasha Liang, Linghu Zeng, Rongchun Huang, Nongqi Yu, Guangsheng Qin, Qiuming Chen, Shengfei Yan

**Affiliations:** 1 Guangxi Buffalo Research Institute, Chinese Academy of Agricultural Sciences, Nanning 530001, Chinaxjau@huangjiajie.top (J.H.);; 2 College of Animal Science, Xinjiang Agricultural University, Urumqi 830052, China

**Keywords:** water buffalo, gastrointestinal microbiome, shotgun metagenomics, gastrointestinal segment, gene-encoded functional profiles, CAZyme

## Abstract

Gastrointestinal segments in ruminants differ in physiological environment and substrate availability. This study characterized taxonomic and gene-encoded functional profiles in 54 shotgun metagenomes from 30 growing male water buffaloes assigned to five dietary formulations. The dataset comprised 30 rumen-fluid samples, six per group, and 24 post-slaughter samples from six gastrointestinal sites in five animals. Comparisons among groups A–E yielded *p* = 0.9816 for final body weight, *p* = 0.9315 for overall average daily gain, and *p* = 0.2832 for rumen-fluid pH. Genus-level principal coordinate analysis (PCoA) used Bray–Curtis dissimilarities calculated from Hellinger-transformed relative abundances. In the 30 rumen-fluid samples, PERMANOVA yielded R^2^ = 0.1554, pseudo-*F* = 1.1499, and *p* = 0.327. The cross-site profiles were described separately from the dietary-group comparison. Post-slaughter rumen contents had larger proportions of Bacteroidota than the other sampled sites, whereas Bacillota accounted for the largest proportions in the cecum and colon. Kyoto Encyclopedia of Genes and Genomes (KEGG) Level 2 profiles and carbohydrate-active enzyme (CAZyme) profiles described the distribution of annotated functions across sites. These data characterize taxonomic assignments and carbohydrate-utilization-related gene profiles from the rumen to the colon in growing water buffaloes.

## 1. Introduction

The ruminant gastrointestinal tract comprises multiple segments that differ in physicochemical conditions, substrate composition, and digestive functions, and each segment supports a distinct microbial environment. Gastrointestinal microorganisms participate in the degradation and fermentation of structural carbohydrates and in microbial protein formation. Previous studies have linked the taxonomic composition and functional characteristics of rumen microorganisms with energy-harvesting efficiency and production performance [1,2]; host-transcriptome studies have also examined links between the rumen microbiome and feed efficiency [3]. Metagenome-assembled genome (MAG) and single-cell studies have further characterized microbial genes and CAZyme resources involved in fiber and complex polysaccharide utilization [4,5].

High-resolution metagenomic resources for ruminants initially focused mainly on the rumen, with public catalogs expanding from 913 rumen microbial genomes to 4941 MAGs [6,7]. As research expanded from the rumen to the entire gastrointestinal tract, a cross-ruminant dataset covered 370 samples from 10 gastrointestinal segments [8], and a buffalo digestive-tract dataset included 695 samples from eight segments [9]. Studies in dairy cattle reported taxonomic and functional differences among the forestomach, small intestine, and hindgut [10,11], while work in beef cattle described the biogeography of microorganisms across gastrointestinal regions [12]. Segment-specific associations with production traits have also been reported: the rumen and cecum harbor different bacterial communities associated with feed efficiency [13], and rumen and hindgut microbiota are linked to average daily gain in calves through different associations [14]. Long-term dietary intervention can alter both rumen and hindgut microbiota together with nutrient degradation across the gastrointestinal tract [15], and microbial community structures in the rumen and hindgut have been separately associated with nitrogen-use efficiency [16].

In recent years, increasing attention has been given to relationships between microorganisms in the lower gastrointestinal tract and feed efficiency, growth, and nitrogen utilization in ruminants [17,18]. Previous work has systematically described microbial resources and spatial distributions throughout the buffalo gastrointestinal tract [8,9]. Dairy- and beef-cattle studies have compared community composition and functional characteristics across anatomical segments [10,11] and extended these comparisons to gastrointestinal microbial biogeography in beef cattle [12]. However, microbiome studies related to nutrition and production in water buffaloes remain centered largely on the rumen, including studies of lactation-stage microbiota and rumen taxonomic and functional characteristics [19,20]. Multi-omics work has further examined links between rumen microorganisms and host metabolism [21], while protein-nutrition and feed-additive trials have also used the rumen as the primary sampling site [22,23]. Within the same feeding trial, applying a consistent taxonomic and functional analysis to the rumen, small intestine, and hindgut of growing water buffaloes allows direct comparison of microbial composition and functions related to carbohydrate utilization across segments. Studies of the ileum and hindgut in cattle have likewise reported associations between segment-specific microorganisms, nutrient utilization, and growth performance [24,25].

This study followed a 150 d feeding schedule with 30 male water buffaloes approximately 12 months of age and used shotgun metagenomic sequencing to describe microbial taxonomic composition and gene-encoded functional characteristics in the rumen, duodenum, jejunum, ileum, cecum, and colon. The analyses included genus-level community structure, major taxa, KEGG Level 2 functional composition, CAZyme classes and family distributions, and correlations between predominant rumen genera and functional features, with a focus on the segment-specific distribution of the gastrointestinal microbiome and functions related to carbohydrate utilization in growing water buffaloes.

## 2. Materials and Methods

### 2.1. Ethics Approval

The animal experiment was approved by the Academic and Animal Ethics Committee of the Guangxi Buffalo Research Institute, Chinese Academy of Agricultural Sciences (approval no. BRI241002; 2 October 2024). All animal feeding, management, and sample-collection procedures were conducted in accordance with institutional animal-welfare requirements.

### 2.2. Animals and Feeding Management

Thirty healthy male water buffaloes approximately 12 months of age and of similar body weight were randomly assigned to five groups (A–E), with six animals per group. The trial was conducted at the Buffalo Breeding Farm of the Guangxi Buffalo Research Institute and was organized as a 150 d feeding schedule in two consecutive stages. Stage 1 lasted 90 d, including a 7 d adaptation period and an 83 d experimental period; Stage 2 lasted 60 d, including a 7 d adaptation period and a 53 d experimental period. The two stages defined the timing of adaptation, digestive–metabolic measurements, and sampling. The same five dietary formulations defined groups A–E in both stages.

Animals were fed a total mixed ration (TMR) once daily and had free access to feed. During the adaptation periods, animal health and adaptation to the diets were monitored. During the experimental periods, the amount of feed offered each day was adjusted according to intake and refusals from the previous day to maintain approximately 10% refusals in the feed bunk. Roughage ingredients and concentrate samples from each group were collected weekly as part of feed monitoring. Concentrates for each group were prepared in batches every 20 d.

### 2.3. Dietary Formulations and Growth Measurements

The ingredient composition and calculated nutrient levels of the five experimental diets are presented on a dry-matter basis. Crude protein concentrations in groups A–E were 13.16%, 14.55%, 15.89%, 17.22%, and 18.58%, respectively. As crude protein increased, the proportion of corn decreased from 24.05% to 0.74%, wheat bran increased from 7.68% to 18.10%, and soybean meal with hulls increased from 3.02% to 16.15%. NDF and phosphorus also increased, whereas ADF and calcium changed little. Corn silage and peanut vine were the main roughage sources (Table 1).

Body weight was measured at baseline, at an intermediate assessment, and at the final assessment. Feed was withdrawn at 20:00 on the day before weighing, and animals were weighed after fasting at approximately 08:00 the following morning. Average daily gain (ADG) was calculated using the nominal 90, 60, and 150 d intervals of the feeding schedule. The 150 d calculation was used as the overall growth measure. Additional body-size measurements, including withers height, chest circumference, and final body length, are summarized by dietary group in Table S2.

### 2.4. Gastrointestinal Sample Collection and Sample Structure

Rumen fluid for metagenomic sequencing was collected during terminal sampling at the end of Stage 2 (day 150), before morning feeding (approximately 07:00 h) using a vacuum-assisted oral rumen tube (outer diameter, 1.5 cm; length, 2.0 m). The initial 10 mL was discarded, and approximately 50 mL of mid-layer fluid was collected and filtered through sterile four-layer gauze. Aliquots were transferred to sterile cryovials, immersed in liquid nitrogen within approximately 30 s, and stored at −80 °C until DNA extraction. Uncentrifuged aliquots were used for metagenomic DNA extraction. Separately, end-of-trial rumen-fluid pH was measured in all 30 buffaloes immediately after collection using a Seven2Go S2 portable pH meter (Mettler-Toledo GmbH, Greifensee, Switzerland). These pH measurements were used for comparisons among groups A–E.

Five buffaloes from the 30-animal trial contributed the post-slaughter multi-segment samples: animal IDs 2315 and 9613 in group A, and 1512, 2339, and 2349 in group C. These animals underwent the final measurements before terminal slaughter sampling. Gastrointestinal contents were collected from the rumen, duodenum, jejunum, ileum, cecum, and colon. Approximately 3 g of contents was transferred from each site to sterile cryovials, frozen in liquid nitrogen, and stored at −80 °C; animal identity and anatomical site were recorded. Sampling concerned luminal contents rather than mucosal tissue.

A total of 54 gastrointestinal samples were included in the metagenomic analysis. The 30 rumen-fluid samples corresponded to the 30 experimental buffaloes, with six samples from each group. The final post-slaughter sequencing set comprised 24 samples: five rumen-content, three duodenal, four jejunal, two ileal, five cecal, and five colonic samples. The remaining collected aliquots were allocated to other planned experimental analyses and were therefore not included in the shotgun metagenomic sequencing set. Rumen-fluid samples defined the A–E comparison, whereas the 24 post-slaughter samples defined the anatomical-site composition summaries. In the 54-sample ordination, rumen fluid and rumen content were displayed as separate sample-source categories. Sample-level metadata are provided in Table S1. For animal IDs 2319 and 2353, the terminal and sequencing labels were used as analytical labels; Table S3 gives their initial and subsequent group-label cross-reference.

### 2.5. Shotgun Metagenomic Sequencing

Total genomic DNA was extracted from gastrointestinal samples using the DNeasy PowerSoil Kit (QIAGEN, Hilden, Germany). After assessment of DNA quality and concentration, samples were used for shotgun metagenomic library construction. Library preparation and sequencing were performed by BGI Genomics (Shenzhen, China).

For each sample, 1 μg of genomic DNA was fragmented by Covaris ultrasonication and size-selected with magnetic beads, yielding fragments mainly between 200 and 400 bp. End repair, 3′-adenylation, adaptor ligation, PCR amplification, and magnetic-bead purification were then performed.

Purified PCR products were denatured and circularized as single-stranded molecules to generate sequencing libraries, and non-circularized linear DNA was removed by digestion. Libraries that passed quality control were sequenced on the DNBSEQ platform with 150 bp paired-end reads (PE150). Raw data were stored in FASTQ format using Phred+33 quality encoding.

### 2.6. Bioinformatic Processing and Taxonomic Annotation

Raw reads were quality-controlled using SOAPnuke v1.5.0 [26] to remove adaptor contamination, reads containing excessive ambiguous bases, and low-quality reads, yielding clean reads. Clean reads were then aligned to the water buffalo (*Bubalus bubalis*) reference genome GCF_019923935.1 (NDDB_SH_1) using Bowtie 2 v2.2.5 [27], and host-derived reads were removed. Unmapped reads were retained for downstream metagenomic analyses.

After host-read removal, clean reads from each sample were assembled de novo using MEGAHIT v1.2.9 [28]. Contigs shorter than 300 bp were removed, and total assembly length, maximum contig length, N50, N90, and mean contig length were calculated. Protein-coding genes were predicted using MetaGeneMark v3.38 [29], and CD-HIT v4.8.1 was used for clustering and redundancy removal to construct a non-redundant gene catalog [30]. Clean reads from each sample were mapped back to the non-redundant gene catalog, and gene relative abundances were estimated using Salmon v1.6.0 [31].

Taxonomic annotation was performed using Kraken 2 v2.1.2 with default parameters (confidence threshold 0.0) [32]. After host-read removal, clean reads were classified against the NCBI NT reference database, and Bracken v2.6.1 was used to re-estimate the relative abundance of each taxon [33]. The resulting abundance tables were used for community-composition analyses and descriptive summaries by sampling source and anatomical site.

### 2.7. Functional Annotation

DIAMOND v0.8.24 was used to align protein sequences encoded by the non-redundant gene catalog against eggNOG v5.0, KEGG v101.0, BacMet (20180311), CARD v3.1.4, COG (20201125), CAZy (20240326), and Swiss-Prot (release-2021_04) [34]. Gene relative abundances estimated with Salmon v1.6.0 [31] were summed for genes sharing the same functional annotation to generate functional-abundance tables for each sample.

The eggNOG database was used for orthologous-group and functional-category annotation [35]. KEGG was used for gene functional annotation [36], and results were summarized at different functional levels. KEGG Level 2 functional composition included carbohydrate metabolism, amino acid metabolism, energy metabolism, glycan biosynthesis and metabolism, membrane transport, and other major categories. Relative abundances of these categories were summarized separately for the 24 post-slaughter samples and the 30 rumen-fluid samples from groups A–E.

CAZyme-related genes were annotated against the CAZy database [37]. Annotations were summarized by class and family, including glycoside hydrolases (GHs), glycosyltransferases (GTs), carbohydrate-binding modules (CBMs), carbohydrate esterases (CEs), polysaccharide lyases (PLs), and auxiliary activities (AAs). Relative abundances of CAZyme classes and families were summarized separately for the 24 post-slaughter samples and the 30 rumen-fluid samples from groups A–E.

### 2.8. Statistical Analysis

Growth-performance and rumen-fluid pH data are presented as mean ± standard deviation, with the individual buffalo used as the experimental unit. Rumen-fluid pH was measured at the end of the trial in all 30 buffaloes, with six animals in each of groups A–E. One-way analysis of variance (ANOVA) was used to compare initial body weight, final body weight, ADG over the nominal 150 d interval, and rumen-fluid pH among groups A–E. *p* < 0.05 was considered statistically significant.

Microbial alpha diversity was described using the genus-level Shannon index with natural logarithms. Each sample in the full genus-abundance table was divided by its column total and square-root transformed before calculation of Bray–Curtis dissimilarities. This Hellinger transformation reduces the weight of highly abundant taxa [38]. Bray–Curtis was then applied to the transformed profiles as an abundance-based dissimilarity. PCoA used the corresponding dissimilarity matrix; axis percentages refer to the sum of positive eigenvalues. Composition plots summarize arithmetic means of within-sample relative abundances, and heatmaps use log10 (relative abundance in percent + 0.001).

PERMANOVA tested the association between community composition and A–E analytical group in the 30 rumen-fluid samples [39]. PERMANOVA used 999 unrestricted label permutations with random seed 20260524; each sample represented a different animal. The 54-sample ordination and the anatomical-site composition summaries were presented descriptively. Shannon indices, individual taxonomic proportions, and KEGG/CAZyme profiles were summarized as descriptive distributions.

**Figure 7:**
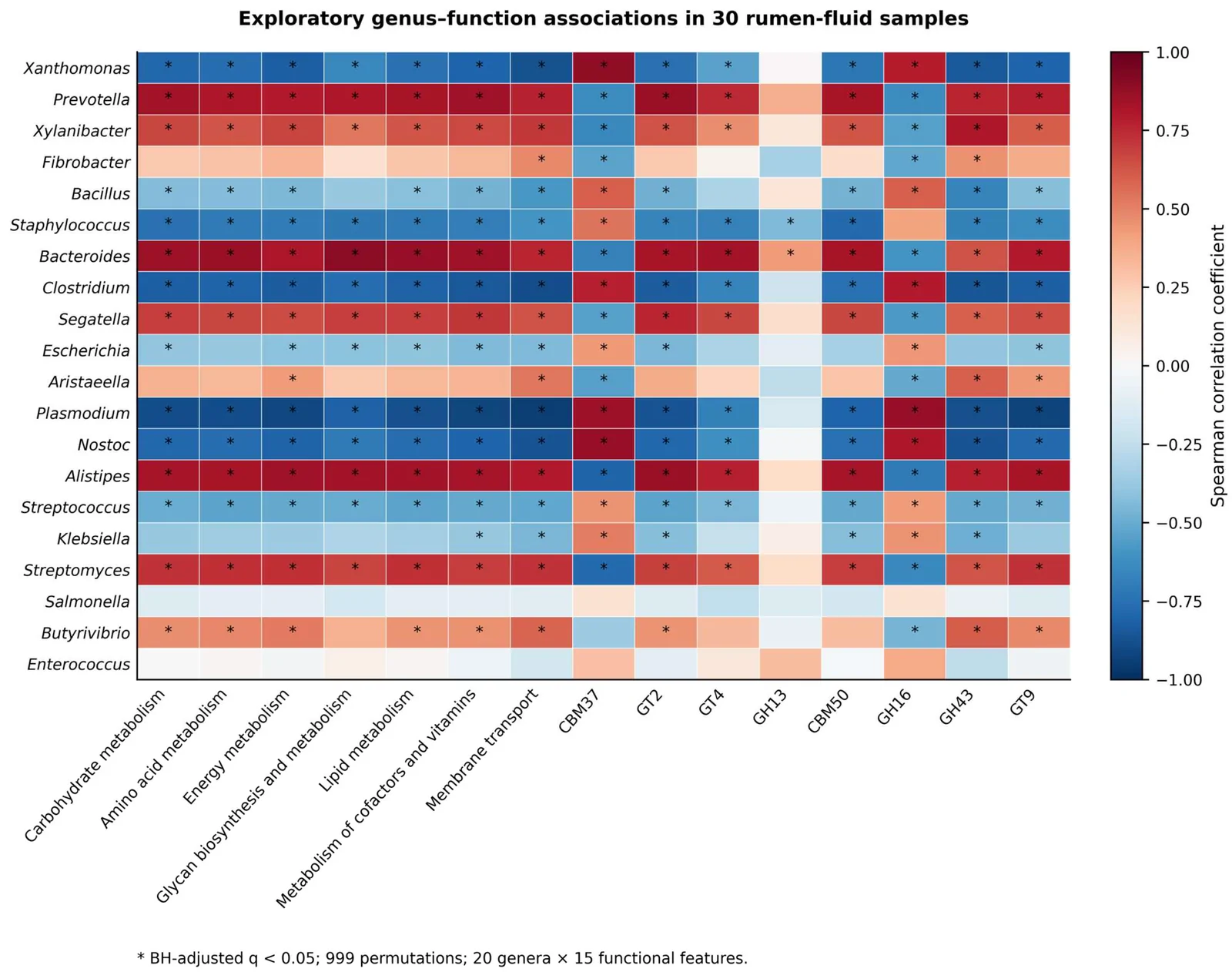
Exploratory genus–function associations in 30 rumen-fluid samples. Rows show the 20 genera with the highest mean abundance; columns show seven KEGG Level 2 categories and eight CAZyme families. Colors represent Spearman coefficients. Asterisks denote *q* < 0.05 after Benjamini–Hochberg correction across 300 pairs; *p* values use 999 two-sided permutations. The figure summarizes co-variation between taxonomic and gene-encoded functional profiles derived from the same metagenomic samples. Pairwise values are provided in Table S5.

Among the 30 rumen-fluid samples, the 20 genera with the highest mean relative abundance were selected for Spearman rank-correlation analysis with the 15 functional features shown in Figure 7. These features comprised seven KEGG Level 2 categories and the eight CAZyme families with the highest mean abundance in the rumen-fluid dataset. Correlation *p* values were obtained from 999 two-sided permutations and adjusted across all 300 pairs using the Benjamini–Hochberg procedure; *q* < 0.05 defined the plotted asterisks. All pairwise coefficients, *p* values, and *q* values are provided in Table S5.

Generative AI was used only for English-language revision.

## 3. Results

### 3.1. Growth Performance and Dietary Composition

**Figure 1:**
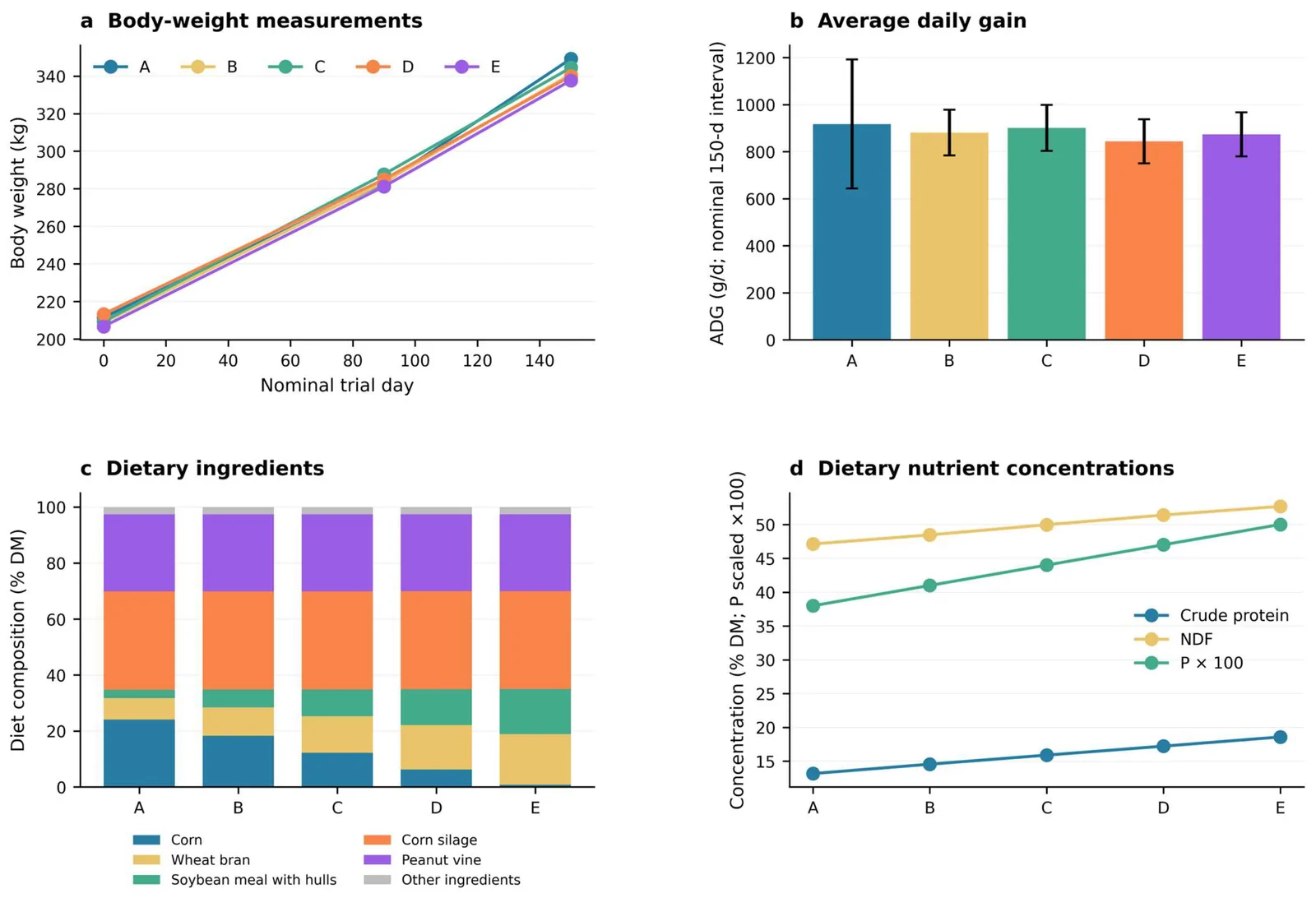
Growth measurements and dietary composition. (**a**) Body weight at the nominal measurement points; (**b**) ADG over the nominal 150 d interval, with mean ± standard deviation; (**c**) major ingredient proportions; (**d**) crude protein (CP), neutral detergent fiber (NDF), and phosphorus (P). Phosphorus is multiplied by 100 for display. Groups A–E are defined in Table 1.

All 30 buffaloes gained weight during the feeding schedule (Figure 1a). The A–E comparisons yielded *p* = 0.9937 for initial body weight and *p* = 0.9816 for final body weight (Table 2).

For ADG over the nominal 150 d interval, the A–E comparison yielded *p* = 0.9315 (Figure 1b; Table 2). Mean rumen-fluid pH ranged from 7.4 to 7.5, with *p* = 0.2832 for the group comparison (Table 2).

From group A to group E, the proportion of corn decreased from 24.05% to 0.74%, wheat bran increased from 7.68% to 18.10%, and soybean meal with hulls increased from 3.02% to 16.15%. The proportions of corn silage and peanut vine changed little, while mineral additives and premix remained constant (Figure 1c; Table 1).

NDF increased from 47.12% to 52.67% and phosphorus from 0.38% to 0.50%; ADF ranged from 30.02% to 30.22% and calcium from 1.36% to 1.41% (Figure 1d; Table 1). The five dietary formulations therefore varied concurrently in crude protein, grain by-products, fiber, and phosphorus.

### 3.2. Sequencing Data and Assembly

**Figure 2:**
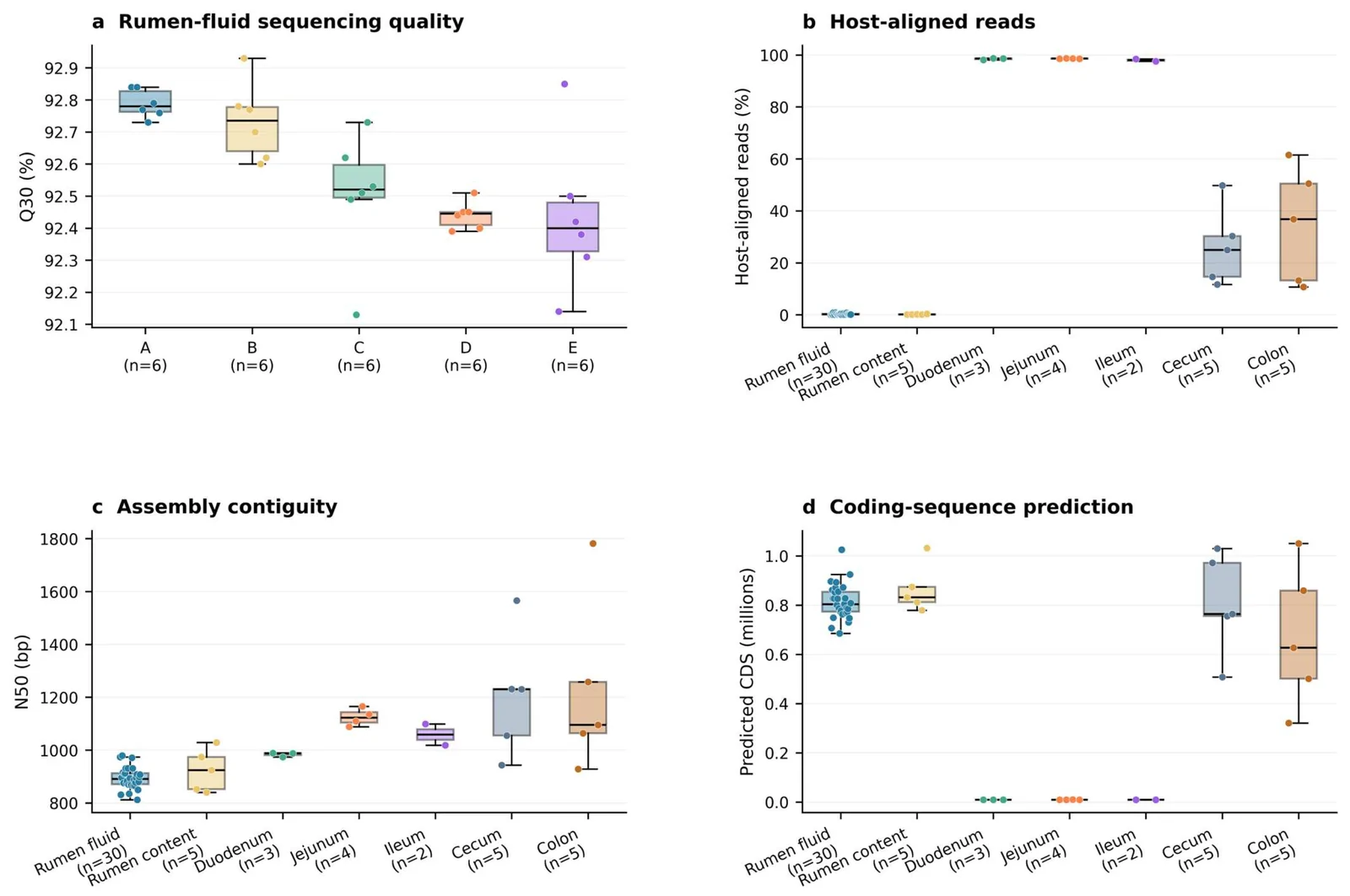
Sequencing and assembly metrics. (**a**) Q30 in 30 rumen-fluid samples from groups A–E (n = 6 each); (**b**) host-read proportion; (**c**) assembly N50; (**d**) predicted coding-sequence (CDS) count. Panels (**b**–**d**) show all 54 samples in seven source categories, with rumen fluid (n = 30) and post-slaughter rumen content (n = 5) separated. Duodenum, n = 3; jejunum, n = 4; ileum, n = 2; cecum and colon, n = 5 each. Dots represent individual samples. Boxes show the interquartile range and median; whiskers extend to 1.5 times the interquartile range.

Across the 30 rumen samples, mean raw-read counts ranged from 210.7 to 213.1 million reads and clean-read counts from 200.5 to 200.7 million reads (Figure 2a; Table 3). Mean Q30 values for groups A, B, C, D, and E were 92.79 ± 0.04%, 92.73 ± 0.12%, 92.50 ± 0.20%, 92.44 ± 0.04%, and 92.43 ± 0.24%, respectively, all above 92%; mean GC content ranged from 35.94% to 39.48%.

Group-mean host-read proportions in rumen fluid ranged from 0.2% to 0.4%, leaving 199.8–200.3 million reads after host removal (Table 3). Across individual small-intestinal samples, host-read proportions ranged from 97.56% to 98.73%, and 2.55–4.90 million reads remained after host removal (Figure 2b; Table S4). The corresponding host-read proportions ranged from 11.74% to 49.81% in cecal samples and from 10.72% to 61.53% in colonic samples. Table S4 reports the sequencing and assembly metrics for all 54 samples.

Assembly N50 ranged from 812 to 979 bp in rumen-fluid samples, from 840 to 1029 bp in post-slaughter rumen contents, and from 974 to 1165 bp in small-intestinal samples. Cecal and colonic N50 values ranged from 943 to 1566 bp and from 928 to 1782 bp, respectively (Figure 2c; Table S4).

Predicted CDS counts ranged from 684,762 to 1,025,279 in rumen-fluid samples and from 779,125 to 1,031,885 in post-slaughter rumen contents. Small-intestinal samples contained 9147–9839 predicted CDSs; cecal and colonic samples contained 507,944–1,029,643 and 320,491–1,050,357, respectively (Figure 2d; Table S4).

### 3.3. Taxonomic Composition

**Figure 3:**
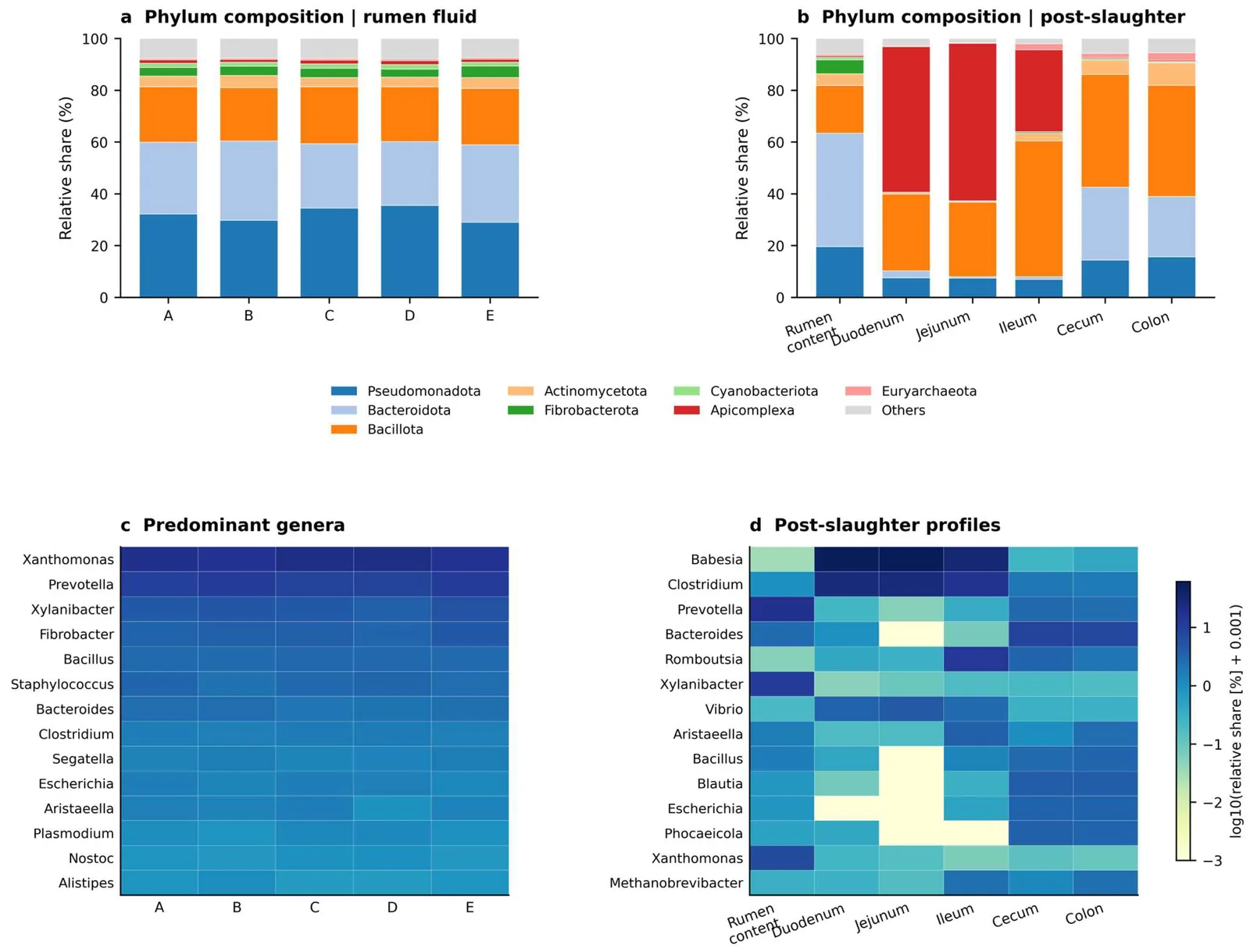
Taxonomic composition of the two sample sets. (**a**,**c**) Rumen-fluid samples from groups A–E, n = 6 per group; (**b**,**d**) post-slaughter samples from rumen content (n = 5), duodenum (n = 3), jejunum (n = 4), ileum (n = 2), cecum (n = 5), and colon (n = 5). Panels (**a**,**b**) show mean phylum proportions, with identical colors for the same phylum. Panels (**c**,**d**) show the 14 genera ranked highest within each comparison, displayed as log10 (mean relative abundance in percent + 0.001) with a common color scale. Taxonomic names refer to metagenomic assignments.

In the 30 rumen-fluid samples, *Pseudomonadota*, *Bacteroidota*, and *Bacillota* accounted for the largest group-mean phylum proportions. Their proportions across groups A–E ranged from 29.0% to 35.5%, 24.6% to 30.7%, and 20.6% to 22.0%, respectively (Figure 3a).

In the 24 post-slaughter samples, *Bacteroidota* accounted for 43.8% of the mean rumen-content profile, followed by *Pseudomonadota* (19.6%) and *Bacillota* (18.4%). Apicomplexa assignments occupied large proportions of the duodenal and jejunal profiles. Bacillota accounted for 52.4% in the ileum, 43.6% in the cecum, and 43.0% in the colon (Figure 3b).

In rumen fluid, *Xanthomonas* and *Prevotella* had the highest mean genus-level assigned proportions, followed by *Xylanibacter* and *Fibrobacter*. *Bacillus*, *Staphylococcus*, *Bacteroides*, *Clostridium*, and *Segatella* were also among the displayed genera (Figure 3c).

The post-slaughter rumen-content profile included *Prevotella* and *Xylanibacter* among the larger assigned genus proportions. *Babesia* and *Clostridium* occupied large proportions of the duodenal and jejunal profiles, whereas *Clostridium* and *Romboutsia* were prominent in the ileal profile. *Bacteroides* accounted for larger mean proportions in the cecum and colon than in rumen contents (Figure 3d).

### 3.4. Alpha Diversity and Community Structure

**Figure 4:**
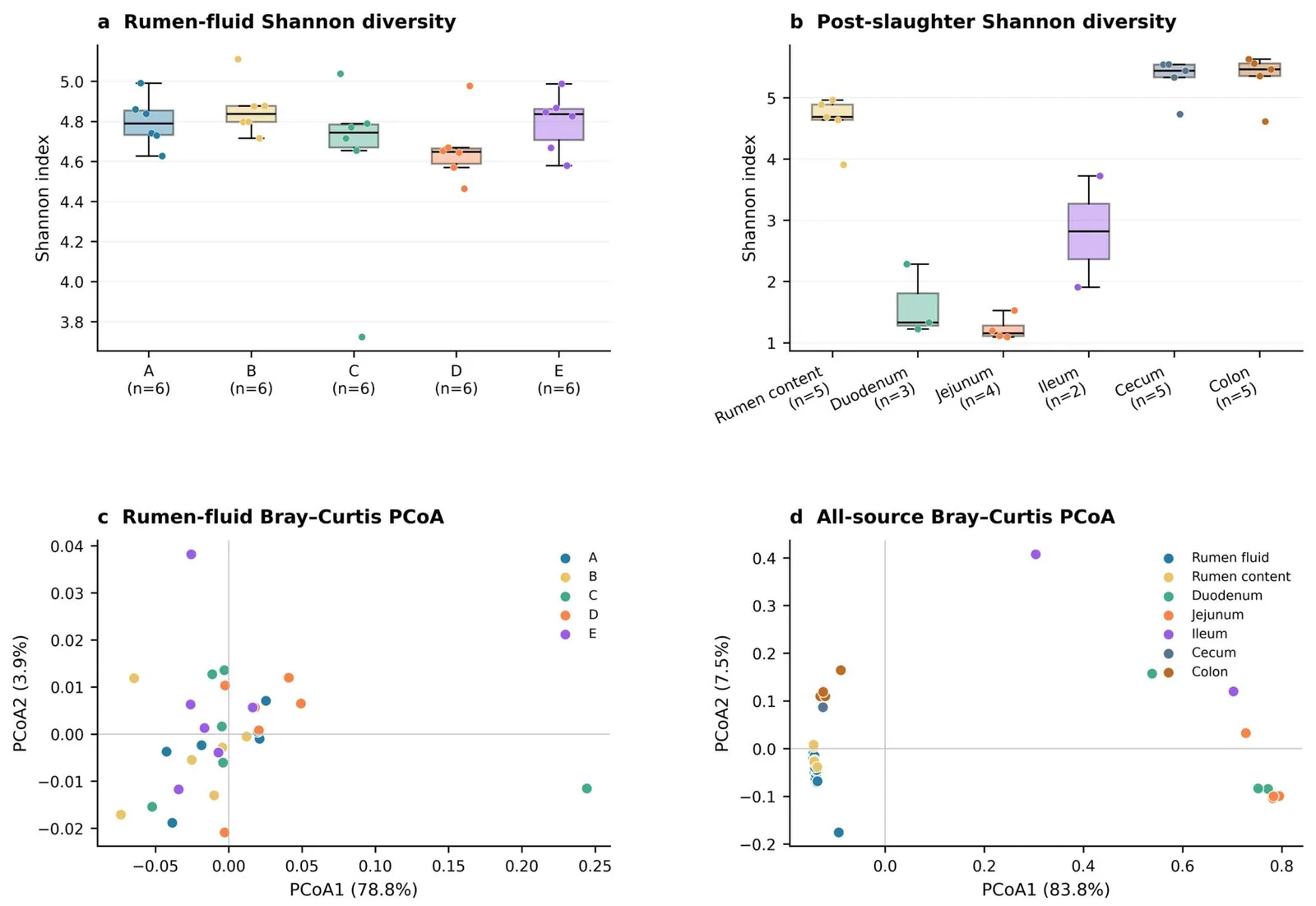
Genus-level Shannon distributions and principal coordinate analysis (PCoA). (**a**) Shannon index in 30 rumen-fluid samples; (**b**) Shannon index in 24 post-slaughter samples; (**c**) PCoA calculated separately for the 30 rumen-fluid samples; (**d**) descriptive PCoA of all 54 samples, distinguishing rumen fluid from rumen content. PCoA uses Bray–Curtis dissimilarities of Hellinger-transformed full genus profiles. Axis percentages are fractions of positive eigenvalue inertia. Each point represents one sample. Sample counts are shown; box and whisker definitions follow Figure 2.

Shannon index distributions overlapped among rumen-fluid samples from groups A–E. Values ranged from 3.72 to 5.11 across all 30 samples; group C included the minimum value (Figure 4a).

Within the post-slaughter set, Shannon values ranged from 3.91 to 4.96 in rumen contents, from 1.23 to 2.29 in the duodenum, from 1.10 to 1.53 in the jejunum, and from 1.91 to 3.73 in the ileum. Cecal and colonic values ranged from 4.73 to 5.54 and from 4.61 to 5.63, respectively (Figure 4b).

In the PCoA of the 30 rumen-fluid samples, axes 1 and 2 represented 78.8% and 3.9% of the positive eigenvalue inertia, respectively. Samples from groups A–E overlapped in ordination space (Figure 4c). The A–E PERMANOVA yielded R^2^ = 0.1554, pseudo- *F* = 1.1499, and *p* = 0.327.

In the descriptive PCoA of all 54 samples, axes 1 and 2 represented 83.8% and 7.5% of the positive eigenvalue inertia. The plot displayed the distribution of samples across seven source categories, including separate rumen-fluid and rumen-content categories (Figure 4d).

### 3.5. KEGG Functional Composition

**Figure 5:**
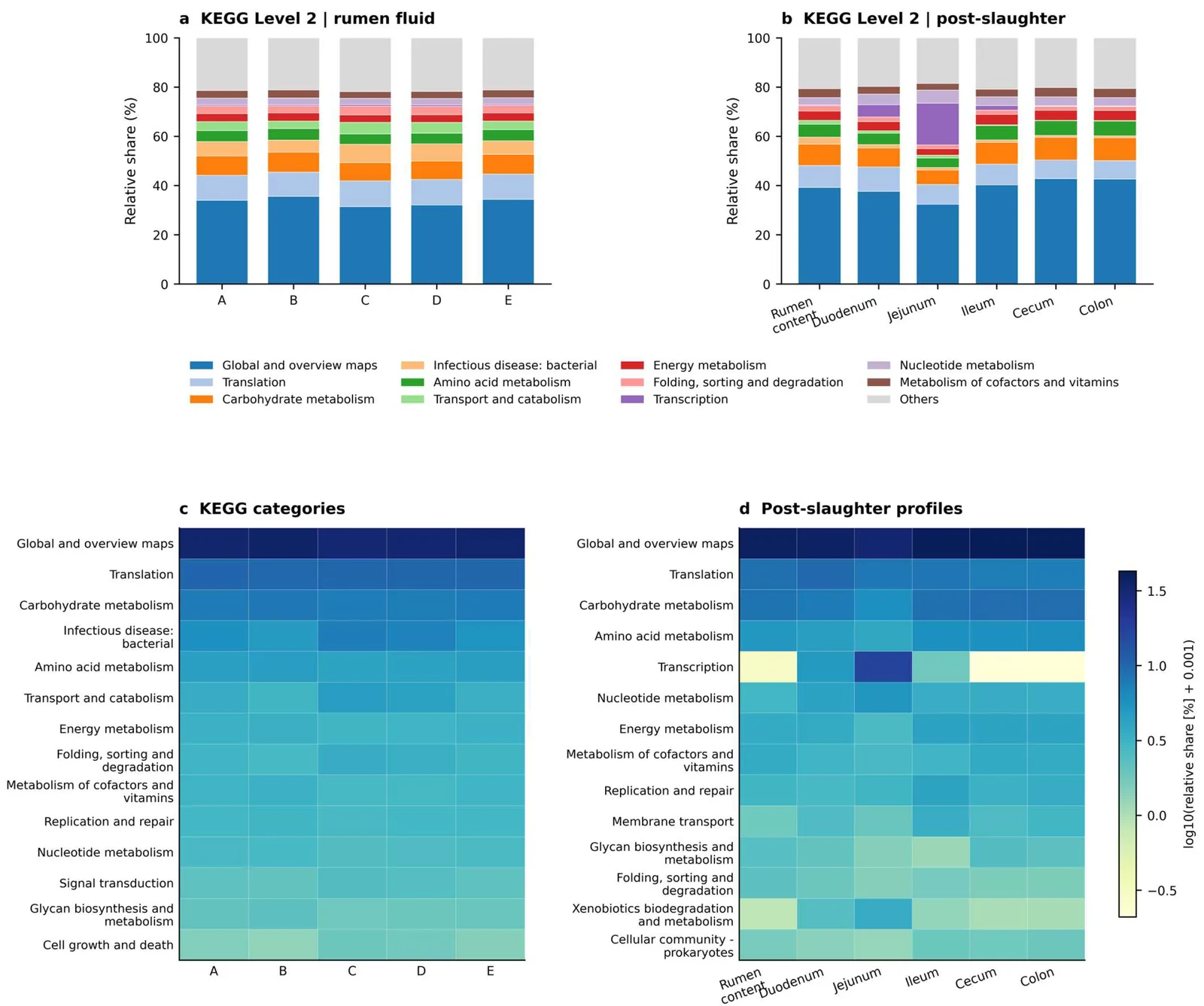
KEGG Level 2 profiles. (**a**,**c**) Thirty rumen-fluid samples summarized by groups A–E (n = 6 each); (**b**,**d**) 24 post-slaughter samples summarized by anatomical site (n = 5, 3, 4, 2, 5, and 5 for rumen content, duodenum, jejunum, ileum, cecum, and colon). Bars show arithmetic means of within-sample annotation proportions. Heatmaps display selected categories as log10 (mean relative abundance in percent + 0.001), using a common color scale. KEGG annotations describe gene-encoded functional profiles.

Across groups A–E, global and overview maps accounted for the largest mean share of the rumen-fluid KEGG Level 2 profile, ranging from 31.5% to 35.6%. Carbohydrate metabolism, translation, and amino acid metabolism were also among the major categories (Figure 5a).

In the post-slaughter set, the mean share of global and overview maps was 39.2% in rumen contents, 37.6% in the duodenum, 32.4% in the jejunum, 40.3% in the ileum, 42.8% in the cecum, and 42.7% in the colon (Figure 5b). These values describe the relative distribution of KEGG annotations within each sample.

The rumen-fluid heatmap displayed global and overview maps, translation, carbohydrate metabolism, and amino acid metabolism among the larger annotated categories. Energy metabolism, metabolism of cofactors and vitamins, replication and repair, and nucleotide metabolism were also represented (Figure 5c).

In the post-slaughter heatmap, carbohydrate metabolism accounted for mean proportions of 8.6% in rumen contents, 7.7% in the duodenum, 5.9% in the jejunum, 8.9% in the ileum, 9.3% in the cecum, and 9.3% in the colon. Amino acid metabolism and energy metabolism were represented at each site (Figure 5d).

### 3.6. CAZyme Composition

**Figure 6:**
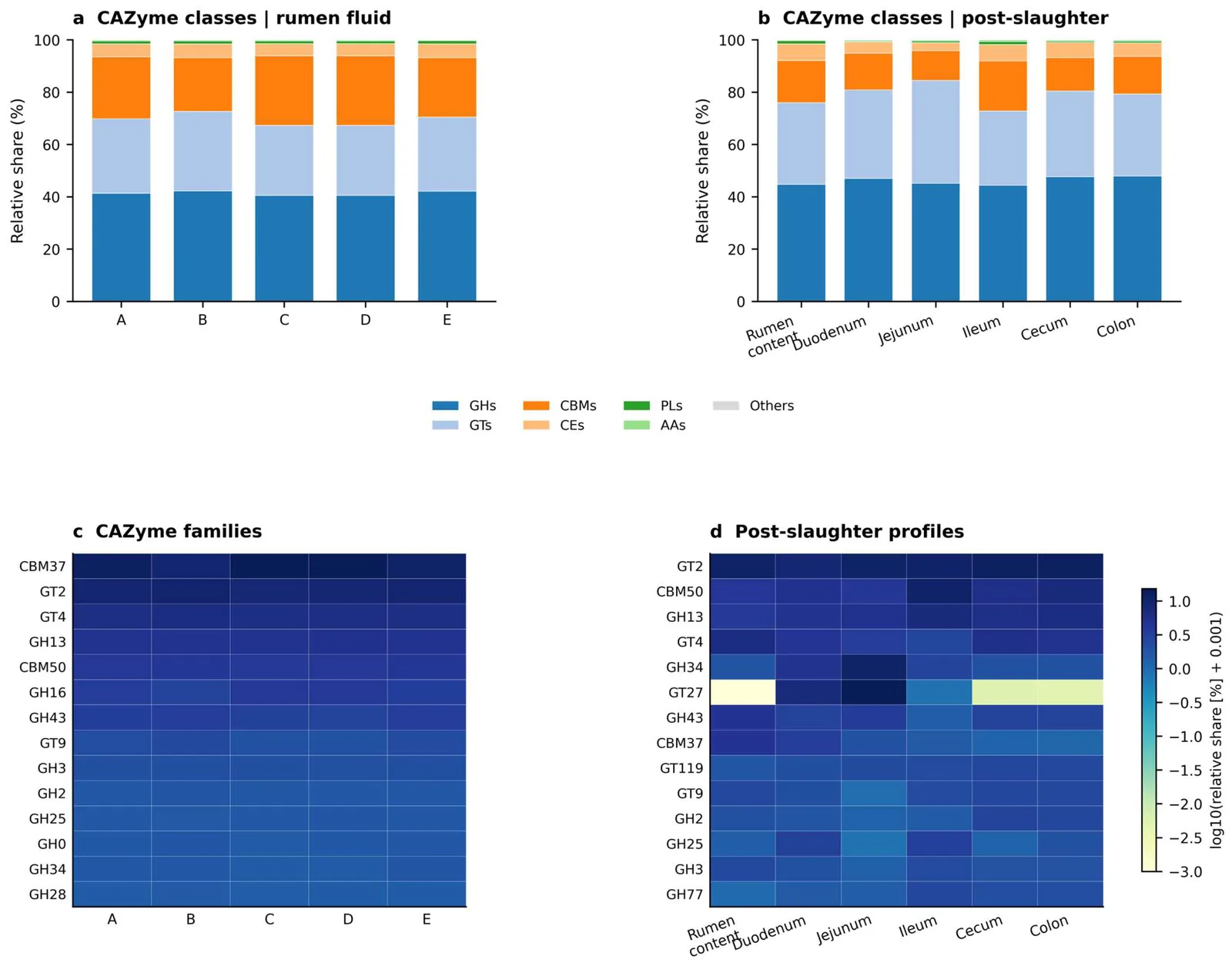
CAZyme profiles. (**a**,**c**) Thirty rumen-fluid samples from groups A–E (n = 6 each); (**b**,**d**) 24 post-slaughter samples from the six anatomical sites, with sample counts as in Figure 5. Panels (**a**,**b**) show mean class proportions; panels (**c**,**d**) show the highest-ranked families as log10 (mean relative abundance in percent + 0.001) with a common color scale. GH, glycoside hydrolase; GT, glycosyltransferase; CBM, carbohydrate-binding module; CE, carbohydrate esterase; PL, polysaccharide lyase; AA, auxiliary activity.

Rumen-fluid samples from groups A–E had the largest class proportions assigned to GHs, GTs, and CBMs, whereas CEs, PLs, and AAs were less abundant (Figure 6a). The class order was GHs, GTs, CBMs, CEs, PLs, and AAs across groups A–E.

Among post-slaughter samples, GHs accounted for mean proportions of 44.5–47.9%, GTs for 28.4–39.4%, and CBMs for 11.3–19.1%. The rumen-content profile comprised 44.7% GHs, 31.3% GTs, and 16.1% CBMs (Figure 6b).

The highest-ranked CAZyme families in rumen fluid included CBM37, GT2, GT4, GH13, CBM50, GH16, GH43, GT9, GH3, and GH2. CBM37 had the largest mean proportion in four groups, whereas GT2 had the largest proportion in group B (Figure 6c).

GT2, CBM50, GH13, and GT4 were among the displayed families across post-slaughter sites. CBM37 occupied a larger mean proportion in rumen contents than in the cecum and colon, whereas GH13 occupied larger proportions in the ileum, cecum, and colon than in rumen contents (Figure 6d). These summaries describe the distribution of family-level annotations.

### 3.7. Species-Level Annotation

Species-level taxonomic assignments for the 30 rumen-fluid samples and the 24 post-slaughter samples are presented separately in Figure S1. The panels display the 20 highest-ranked species within each sample-set summary.

### 3.8. Exploratory Genus–Function Associations

Spearman rank correlations between the 20 predominant genera and 15 functional features are shown in Figure 7. The 15 features included seven KEGG Level 2 categories and eight CAZyme families, yielding 300 genus–function combinations. With 999 permutations per pair and Benjamini–Hochberg correction, 218 combinations had *q* < 0.05, with a minimum *q* value of 0.0021.

*Bacteroides* and *Alistipes* had positive Spearman coefficients with the seven displayed KEGG categories and with GT2, GT4, CBM50, GH43, and GT9. Their coefficients with CBM37 and GH16 were negative. *Prevotella* also had positive coefficients with the seven KEGG categories and with GT2, GT4, CBM50, GH43, and GT9 (Figure 7; Table S5).

*Clostridium* had negative coefficients with the seven KEGG categories and positive coefficients with CBM37 and GH16. For *Fibrobacter*, coefficients were positive for membrane transport and GH43 and negative for CBM37 and GH16 (Figure 7). These coefficients summarize within-dataset co-variation.

## 4. Discussions

This study characterized shotgun metagenomic profiles from the rumen to the colon in growing water buffaloes. The 30 rumen-fluid samples overlapped with A–E groups in PCoA space, with PERMANOVA *p* = 0.327. The post-slaughter profiles described variations in taxonomic assignments and KEGG/CAZyme composition across the six sampled sites. This anatomical distribution can be compared with the buffalo gastrointestinal resource described by Tong et al. [9] and the region-specific communities reported in dairy cattle [11].

*Prevotella* and *Xylanibacter* occupied larger assigned proportions in post-slaughter rumen contents than in the hindgut, whereas *Bacteroides* occupied larger proportions in cecal and colonic contents. These profiles concern luminal communities, complementing studies of cattle gastrointestinal mucosa [12]. Rumen and cecal communities have also been studied separately in relation to feed efficiency [13], and dietary interventions can affect rumen and hindgut profiles together [15,16]. The comparisons here distinguish anatomical site from sampling source by retaining oral rumen fluid and post-slaughter rumen contents as separate categories.

GHs, GTs, and CBMs accounted for the largest CAZyme class proportions in both sample sets. Among post-slaughter sites, the CBM37 annotation proportion was larger in rumen contents than in the cecum and colon, while GH13 showed the opposite ordering. According to CAZy classification, CBMs concern carbohydrate binding, GH families include enzymes that hydrolyze glycosidic bonds, and GTs concern glycosyl transfer [37]. Studies of forage-to-concentrate ratio, lignin supply, and rumen-degradable starch have related these substrate contrasts to rumen CAZyme composition [40,41,42]. The present annotations describe the distribution of carbohydrate-related gene families along the gastrointestinal tract. Buffalo studies have likewise reported diet-associated shifts in expressed functional genes and rumen functional profiles [43,44]. Related metagenomic work indicates that nitrogen supply and lignin-rich substrates can modify microbial or carbohydrate-active profiles [45,46].

KEGG annotations included categories for carbohydrate, amino-acid, and energy metabolism at all sampled sites. These categories represent gene-encoded potential rather than measured reaction rates. The rumen-only correlation analysis related genus profiles to seven KEGG categories and eight CAZyme families. Positive coefficients for *Bacteroides*, *Alistipes*, and *Prevotella* with several displayed categories describe co-variation within this dataset. Metatranscriptomic and multi-omics studies have linked active rumen functions with feed efficiency and host performance [47,48]. Heritable carbohydrate-metabolism features and diet-associated functional shifts have also been identified in cattle [49,50]. Metagenomic functional patterns vary with methane phenotype and residual feed intake, underscoring that such functional signatures are context-dependent [51,52].

The five dietary formulations varied concurrently in corn, wheat bran, soybean meal with hulls, crude protein, and NDF. The 5.42-percentage-point crude-protein range therefore accompanied changes in starch- and fiber-supplying ingredients. Rumen microbial associations with feed efficiency can depend on dietary composition [53], and forage-to-concentrate contrasts can modify fermentation and nutrient digestion [54,55]. In vitro work by İnanç et al. also examined TMRs differing in forage-to-concentrate ratio together with rutin and monensin [56]. Its batch-culture setting provides a dietary-substrate comparison rather than a direct test of the present in vivo treatments. Here, the A–E tests gave *p* = 0.9816 for final body weight, *p* = 0.9315 for overall ADG, and *p* = 0.327 for rumen-fluid community composition. These results describe the responses under the combined formulation contrasts rather than an isolated crude-protein effect. Such context dependence is also evident in broad diet–host comparisons and in protein restriction followed by re-alimentation [57,58]. Breed-associated metagenomic differences and integrated feed-efficiency studies further indicate that host background can modify microbial associations [59,60]. Rumen and fecal responses may also diverge across breed–diet combinations and during age-related dietary transitions [61,62].

These segment-resolved profiles should also be viewed in the context of studies showing that downstream communities can respond differently from the rumen during feed-additive exposure and weaning [63,64], while concentrate-rich feeding can affect both reticulorumen and hindgut fermentation [65]. Variation linked to age and host genetics provides an additional source of heterogeneity across animals [66,67], and associations between rumen communities and feed efficiency can differ between postweaning and finishing diets [68]. Comparative cattle–buffalo metagenomics has related rumen CAZyme profiles to methane yield and fermentation [69], whereas dietary energy and rumen multi-omics have been related to growth-associated phenotypes [70,71]. Genome-centric studies from rumen and fecal samples provide additional context for interpreting bovine gastrointestinal metagenomes [72,73].

The group-mean rumen-fluid pH values were 7.4–7.5. Oral-tube sampling and sampling position can influence pH relative to fluid collected through a cannula [74]. The initial aspirate was discarded in the present collection procedure; interpretation nevertheless concerns pre-feeding oral-tube samples. The pH comparison is therefore specific to that collection context. Saliva carryover during oral-tube sampling may also contribute to elevated absolute pH values [74].

Several considerations define the scope of the segment-level microbial profiles presented here. Host-derived reads accounted for a high proportion of the sequencing data in the small-intestinal samples; nevertheless, 2.55–4.90 million non-host reads remained after host-read removal and were available for downstream taxonomic and functional profiling. The present study focused on luminal contents and therefore characterizes microbial communities associated with digestive contents rather than mucosa-associated communities. Several database-derived taxonomic assignments highlighted in the review, including *Babesia*, *Plasmodium*, *Xanthomonas*, and *Clostridium botulinum*, are interpreted at the annotation level rather than as independent confirmation of organism presence; their interpretation depends on the reference database and the sequence pool available after host-read removal [75,76]. Because taxonomic and functional profiles were derived from the same metagenomic data, the genus–function correlations are treated as within-dataset co-variation rather than mechanistic associations. These factors define the scope of the segment-level comparisons presented here, particularly for the small intestine. The post-slaughter set comprised repeated anatomical samples from five animals, and the cross-site ordination and compositional summaries were therefore interpreted descriptively.

## 5. Conclusions

This study characterized taxonomic assignments and gene-encoded functional profiles in 30 rumen-fluid samples and 24 post-slaughter gastrointestinal-content samples from growing water buffaloes. The site-resolved summaries described phylum and genus composition together with KEGG Level 2 categories and CAZyme classes and families. Carbohydrate-utilization-related annotations were represented from the rumen to the colon. The dataset provides descriptive information on luminal metagenomic profiles across the sampled gastrointestinal sites.

## Figures and Tables

**Table 1:** Ingredient composition and calculated nutrient levels of the five dietary formulations (dry-matter basis).

Item	Group A	Group B	Group C	Group D	Group E
Corn (%)	24.05	18.30	12.19	6.27	0.74
Wheat bran (%)	7.68	10.10	13.08	15.87	18.10
Soybean meal with hulls (%)	3.02	6.40	9.59	12.78	16.15
Dicalcium phosphate (%)	0.60	0.60	0.60	0.60	0.60
Sodium bicarbonate (%)	0.56	0.56	0.56	0.56	0.56
Salt (%)	0.41	0.41	0.41	0.41	0.41
Limestone (%)	0.41	0.41	0.41	0.41	0.41
Mycotoxin binder (%)	0.18	0.18	0.18	0.18	0.18
Premix (%)	0.40	0.40	0.40	0.40	0.40
Corn silage (%)	35.16	35.13	35.10	35.06	35.03
Peanut vine (%)	27.52	27.50	27.47	27.45	27.42
Total (%)	100.00	100.00	100.00	100.00	100.00
Crude protein (%)	13.16	14.55	15.89	17.22	18.58
NDF (%)	47.12	48.46	49.96	51.39	52.67
ADF (%)	30.02	30.06	30.13	30.18	30.22
Ca (%)	1.36	1.37	1.38	1.40	1.41
P (%)	0.38	0.41	0.44	0.47	0.50

Note: Nutrient concentrations are formulation-derived values on a dry-matter basis. Groups A–E correspond to the five dietary formulations containing 13.16%, 14.55%, 15.89%, 17.22%, and 18.58% crude protein, respectively. NDF, neutral detergent fiber; ADF, acid detergent fiber.

**Table 2:** Growth performance and rumen-fluid pH of growing water buffaloes fed the five dietary formulations.

Variable	Group A	Group B	Group C	Group D	Group E	*p* Value
Initial body weight (kg)	211.5 ± 27.0	208.8 ± 22.4	209.4 ± 31.7	213.3 ± 22.8	206.7 ± 27.0	0.9937
Intermediate body weight (kg)	283.0 ± 38.5	283.5 ± 22.8	287.7 ± 36.6	285.0 ± 30.2	281.2 ± 25.0	
Final body weight (kg)	349.2 ± 51.4	341.0 ± 18.6	344.5 ± 38.7	340.0 ± 30.7	337.7 ± 24.9	0.9816
ADG, nominal days 0–90 (g/d)	794.4 ± 161.0	829.6 ± 130.5	869.4 ± 98.9	796.3 ± 106.5	827.8 ± 65.1	
ADG, nominal days 90–150 (g/d)	1102.8 ± 642.1	958.3 ± 105.3	947.2 ± 159.0	916.7 ± 156.3	941.7 ± 224.8	
ADG, nominal days 0–150 (g/d)	917.8 ± 274.2	881.1 ± 97.1	900.6 ± 97.7	844.4 ± 93.7	873.3 ± 93.7	0.9315
Total body-weight gain (kg)	137.7 ± 41.1	132.2 ± 14.6	135.1 ± 14.7	126.7 ± 14.1	131.0 ± 14.1	
Rumen fluid pH	7.5 ± 0.1	7.4 ± 0.1	7.5 ± 0.1	7.4 ± 0.1	7.4 ± 0.1	0.2832

Note: Values are mean ± standard deviation (n = 6 per group). Groups A–E correspond to the five dietary formulations containing 13.16%, 14.55%, 15.89%, 17.22%, and 18.58% crude protein, respectively. Day 0, day 90, and day 150 denote the nominal points in the feeding schedule; the reported ADG values use 90, 60, and 150 d denominators. Rumen-fluid pH was measured in all 30 buffaloes. *p* values were obtained by one-way ANOVA using the individual buffalo as the experimental unit.

**Table 3:** Sequencing quality metrics of the rumen metagenomic samples.

Group	Raw Reads (Million)	Clean Reads (Million)	Q30 (%)	GC (%)	Host-Derived Reads (%)	Clean Reads After Host Removal (Million)
A	211.0 ± 0.7	200.7 ± 0.0	92.79 ± 0.04	37.67 ± 2.40	0.3 ± 0.3	200.1 ± 0.6
B	210.7 ± 0.6	200.7 ± 0.1	92.73 ± 0.12	39.48 ± 2.60	0.3 ± 0.3	200.0 ± 0.6
C	211.4 ± 2.6	200.5 ± 0.1	92.50 ± 0.20	35.97 ± 6.06	0.3 ± 0.2	199.8 ± 0.3
D	211.4 ± 1.3	200.6 ± 0.0	92.44 ± 0.04	35.94 ± 1.68	0.4 ± 0.3	199.9 ± 0.6
E	213.1 ± 1.2	200.6 ± 0.1	92.43 ± 0.24	38.44 ± 1.42	0.2 ± 0.1	200.3 ± 0.2

Note: Values are mean ± standard deviation for the 30 rumen-fluid samples (n = 6 per group). Groups A–E correspond to the five dietary formulations containing 13.16%, 14.55%, 15.89%, 17.22%, and 18.58% crude protein, respectively.

## Data Availability

The metagenomic sequencing data generated in this study are publicly available in the NCBI BioProject database under accession number PRJNA1321136 (https://www.ncbi.nlm.nih.gov/bioproject/PRJNA1321136/, accssed on 28 September 2026).

## Supplementary Materials

The following supporting information can be downloaded at: https://www.mdpi.com/article/10.3390/microorganisms14102206/s1, Figure S1: Species-level assignments in the two sample sets; Table S1: Sample-level metadata; Table S2: Body-size measurements by group; Table S3: Cross-reference of initial and terminal/sample group labels; Table S4: Sequencing and assembly metrics for all 54 samples; Table S5: Rumen-only genus–function correlations.
