# Supplementary material for: Taxonomic and Functional Profiles of the Gastrointestinal Microbiome Across Digestive Tract Segments in Growing Water Buffaloes
Source: Microorganisms. 2026 Oct 1;14(10):2206. doi: 10.3390/microorganisms14102206 (PMC13707840; doi:10.3390/microorganisms14102206)
Supplement: Supplementary file 1 [file microorganisms-14-02206-s001.zip › microorganisms-4569030-supplementary.pdf]

microorganisms

MDPI

Supplementary Materials

Taxonomic and Functional Profiles of the Gastrointestinal Microbiome Across Digestive Tract Segments in Growing Water Buffaloes

Manuscript ID: microorganisms-4569030

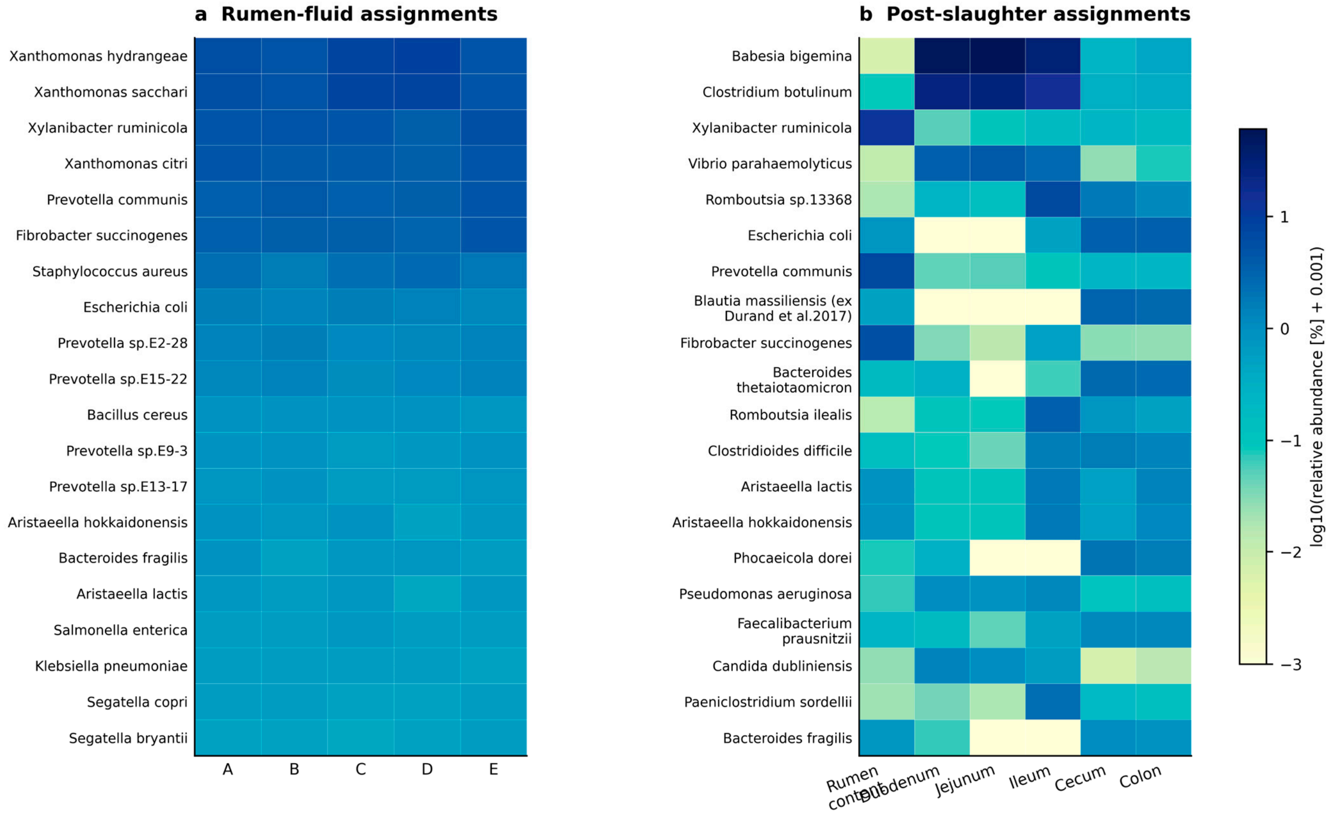

Figure S1. Species-level assignments in the two sample sets. (a) Thirty rumen-fluid samples, summarized by groups A–E (n=6 per group); (b) 24 post-slaughter samples, summarized by rumen content (n=5), duodenum (n=3), jejunum (n=4), ileum (n=2), cecum (n=5), and colon (n=5). Each panel displays the 20 species ranked highest within its corresponding summary. Values are log10(mean relative abundance in percent + 0.001), using a common color scale. Names denote metagenomic taxonomic assignments; their interpretation follows the final Discussion paragraph.

Supplementary Tables

 https://doi.org/10.3390/microorganisms14102206

Table S1. Sample-level metadata

sample_id	animal_id	analytical_group	sample_set	sampling_source	initial_body_weight_kg	final_body_weight_kg	ADG_nominal_150d_g_per_day
A_1520A	1520	A	Rumen-fluid cohort	Rumen fluid	191	294	686.7
A_2277A	2277	A	Rumen-fluid cohort	Rumen fluid	199	413	1426.7
A_2283A	2283	A	Rumen-fluid cohort	Rumen fluid	231	363	880.0
A_2315A	2315	A	Rumen-fluid cohort	Rumen fluid	229	342	753.3
A_2347A	2347	A	Rumen-fluid cohort	Rumen fluid	175	288	753.3
A_9613A	9613	A	Rumen-fluid cohort	Rumen fluid	244	395	1006.7
B_1522A	1522	B	Rumen-fluid cohort	Rumen fluid	197	348	1006.7
B_2317A	2317	B	Rumen-fluid cohort	Rumen fluid	223	335	746.7
B_2319A	2319	B	Rumen-fluid cohort	Rumen fluid	242	373	873.3
B_2343A	2343	B	Rumen-fluid cohort	Rumen fluid	212	331	793.3
B_2351A	2351	B	Rumen-fluid cohort	Rumen fluid	202	341	926.7
B_2373A	2373	B	Rumen-fluid cohort	Rumen fluid	177	318	940.0
C_1512A	1512	C	Rumen-fluid cohort	Rumen fluid	205	330	833.3
C_2323A	2323	C	Rumen-fluid cohort	Rumen fluid	248	396	986.7
C_2337A	2337	C	Rumen-fluid cohort	Rumen fluid	240	385	966.7
C_2339A	2339	C	Rumen-fluid cohort	Rumen fluid	214	329	766.7
C_2349A	2349	C	Rumen-fluid cohort	Rumen fluid	183.5	334	1003.3
C_2353A	2353	C	Rumen-fluid cohort	Rumen fluid	166	293	846.7
D_1524A	1524	D	Rumen-fluid cohort	Rumen fluid	193	313	800.0
D_2297A	2297	D	Rumen-fluid cohort	Rumen fluid	256	384	853.3
D_2325A	2325	D	Rumen-fluid cohort	Rumen fluid	209	344	900.0
D_2331A	2331	D	Rumen-fluid cohort	Rumen fluid	212	356	960.0
D_2357A	2357	D	Rumen-fluid cohort	Rumen fluid	195	298	686.7
D_9633A	9633	D	Rumen-fluid cohort	Rumen fluid	215	345	866.7
E_1506A	1506	E	Rumen-fluid cohort	Rumen fluid	215	347	880.0
E_2307A	2307	E	Rumen-fluid cohort	Rumen fluid	246	372	840.0
E_2327A	2327	E	Rumen-fluid cohort	Rumen fluid	190	320	866.7
E_2329A	2329	E	Rumen-fluid cohort	Rumen fluid	219	328	726.7
E_2375A	2375	E	Rumen-fluid cohort	Rumen fluid	167	304	913.3
E_9611A	9611	E	Rumen-fluid cohort	Rumen fluid	203	355	1013.3
CE_A2315A	2315	A	Post-slaughter cohort	Cecum	229	342	753.3
CE_A9613A	9613	A	Post-slaughter cohort	Cecum	244	395	1006.7
CE_C1512A	1512	C	Post-slaughter cohort	Cecum	205	330	833.3
CE_2339A	2339	C	Post-slaughter cohort	Cecum	214	329	766.7
CE_C2349A	2349	C	Post-slaughter cohort	Cecum	183.5	334	1003.3
CO_A2315B	2315	A	Post-slaughter cohort	Colon	229	342	753.3
CO_A9613A	9613	A	Post-slaughter cohort	Colon	244	395	1006.7
CO_C1512A	1512	C	Post-slaughter cohort	Colon	205	330	833.3
CO_2339A	2339	C	Post-slaughter cohort	Colon	214	329	766.7
CO_C2349A	2349	C	Post-slaughter cohort	Colon	183.5	334	1003.3
DM_A2315A	2315	A	Post-slaughter cohort	Duodenum	229	342	753.3
DM_A9613A	9613	A	Post-slaughter cohort	Duodenum	244	395	1006.7
DM_2339A	2339	C	Post-slaughter cohort	Duodenum	214	329	766.7

sample_id	animal_id	analytical_group	sample_set	sampling_source	initial_body_weight_kg	final_body_weight_kg	ADG_nominal_150d_g_per_day
IM_A2315A	2315	A	Post-slaughter cohort	Ileum	229	342	753.3
IM_A9613A	9613	A	Post-slaughter cohort	Ileum	244	395	1006.7
JM_A2315A	2315	A	Post-slaughter cohort	Jejunum	229	342	753.3
JM_A9613A	9613	A	Post-slaughter cohort	Jejunum	244	395	1006.7
JM_C1512A	1512	C	Post-slaughter cohort	Jejunum	205	330	833.3
JM_C2349A	2349	C	Post-slaughter cohort	Jejunum	183.5	334	1003.3
R_A2315A	2315	A	Post-slaughter cohort	Rumen content	229	342	753.3
R_A9613A	9613	A	Post-slaughter cohort	Rumen content	244	395	1006.7
R_C1512A	1512	C	Post-slaughter cohort	Rumen content	205	330	833.3
R_2339A	2339	C	Post-slaughter cohort	Rumen content	214	329	766.7
R_C2349A	2349	C	Post-slaughter cohort	Rumen content	183.5	334	1003.3

Note: The 54 rows link sample ID, animal ID, analytical group, sample set, sampling source, and the reported growth measurements. Rumen fluid denotes the 30 A–E samples. Rumen content and the five intestinal sites denote the 24 post-slaughter samples. ADG entries retain the nominal 150-d denominator used in the manuscript.

Table S2. Body-size measurements by group

Group	Initial withers height (cm)	Final withers height (cm)	Initial chest circumference (cm)	Final chest circumference (cm)	Final body length (cm)
A	112.0 ± 4.8	121.7 ± 5.1	149.2 ± 12.7	174.0 ± 9.0	122.7 ± 4.9
B	110.3 ± 2.4	121.0 ± 2.4	144.0 ± 5.2	169.5 ± 3.4	123.7 ± 2.4
C	108.3 ± 4.0	121.8 ± 3.9	143.0 ± 6.7	166.3 ± 5.6	123.5 ± 3.4
D	110.7 ± 4.6	121.5 ± 4.5	142.8 ± 7.2	168.0 ± 4.4	121.7 ± 4.6
E	107.3 ± 4.2	118.5 ± 3.2	140.7 ± 6.6	167.5 ± 6.0	121.5 ± 6.2

Note: Values are group summaries for initial and final withers height, chest circumference, and final body length. Values are mean ± standard deviation in centimeters.

Table S3. Cross-reference of initial and terminal/sample group labels

animal_id	initial_group_label	terminal_group_label	sequencing_sample_id	analytical_group	analytical_label_rule
2319	C	B	B_2319A	B	Terminal/sequencing label used
2353	B	C	C_2353A	C	Terminal/sequencing label used

Note: The table shows the initial and terminal/sequencing group labels for animals 2319 and 2353 and the analytical labels used. The terminal/sequencing labels were used as the analytical labels in the final analysis.

Table S4. Sequencing and assembly metrics for all 54 samples

sample_id	animal_id	analytical_group	sampling_source	raw_reads	clean_reads	post_host_reads	host_reads_removed	host_read_percentage	contig_number	assembly_length_bp	N50_bp	N90_bp	predicted_CDS	redundancy_filtered_CDS	Q30_percentage	GC_percentage
A_1520A	1,520	A	Rumen fluid	212,160,000	200,713,132	200,575,786	137,346	0.07	619,320	527,232,183	974	389	896,060	674,579	92.84	40.34
A_2277A	2,277	A	Rumen fluid	210,480,000	200,718,690	200,271,762	446,928	0.22	524,852	390,663,869	831	358	707,069	498,318	92.84	40.51
A_2283A	2,283	A	Rumen fluid	210,560,000	200,771,234	200,322,982	448,252	0.22	629,106	492,390,110	896	364	861,545	605,887	92.77	38.41

https://doi.org/10.3390/microorganisms14102206

sample_id	animal_id	analytical_group	sampling_source	raw_reads	clean_reads	post_host_reads	host_reads_removed	host_read_percentage	contig_number	assembly_length_bp	N50_bp	N90_bp	predicted_CDS	redundancy_filtered_CDS	Q30_percentage	GC_percentage
A_2315A	2,315	A	Rumen fluid	210,160,000	200,727,318	200,536,386	190,932	0.10	616,538	513,794,763	979	377	827,012	567,207	92.73	35.39
A_2347A	2,347	A	Rumen fluid	211,120,000	200,674,230	199,049,428	1,624,802	0.81	571,707	451,964,384	915	369	749,072	488,474	92.79	35.58
A_9613A	9,613	A	Rumen fluid	211,520,000	200,658,610	200,086,952	571,658	0.28	626,669	482,673,152	876	365	827,259	573,116	92.76	35.82
B_1522A	1,522	B	Rumen fluid	211,440,000	200,792,064	200,543,410	248,654	0.12	591,062	480,963,112	910	372	853,083	646,341	92.78	42.66
B_2317A	2,317	B	Rumen fluid	210,960,000	200,573,852	198,939,796	1,634,056	0.81	643,135	517,076,537	913	375	870,795	619,634	92.93	37.84
B_2319A	2,319	B	Rumen fluid	210,320,000	200,663,672	199,817,336	846,336	0.42	598,811	492,876,435	931	368	892,515	688,208	92.77	42.70
B_2343A	2,343	B	Rumen fluid	210,400,000	200,586,774	199,815,952	770,822	0.38	595,341	458,376,898	876	365	800,205	556,246	92.70	38.23
B_2351A	2,351	B	Rumen fluid	211,200,000	200,727,236	200,487,418	239,818	0.12	628,225	486,600,716	883	366	825,839	550,887	92.60	36.50
B_2373A	2,373	B	Rumen fluid	209,840,000	200,645,544	200,405,462	240,082	0.12	614,639	496,037,182	931	369	855,137	610,130	92.62	38.97
C_1512A	1,512	C	Rumen fluid	210,480,000	200,551,468	199,851,036	700,432	0.35	561,201	443,065,117	870	376	786,537	565,377	92.62	41.07
C_2323A	2,323	C	Rumen fluid	216,560,000	200,418,900	199,795,746	623,154	0.31	522,061	385,601,256	835	359	684,762	465,916	92.49	37.76
C_2337A	2,337	C	Rumen fluid	210,080,000	200,600,316	199,931,648	668,668	0.33	579,203	449,669,408	893	365	773,524	523,331	92.13	37.59
C_2339A	2,339	C	Rumen fluid	211,360,000	200,592,508	199,701,898	890,610	0.44	577,031	447,112,770	871	368	777,573	545,745	92.51	38.00
C_2349A	2,349	C	Rumen fluid	210,320,000	200,332,386	200,226,938	105,448	0.05	864,439	652,657,340	867	371	1,025,279	643,013	92.73	23.91
C_2353A	2,353	C	Rumen fluid	209,760,000	200,411,606	199,358,986	1,052,620	0.53	571,543	472,914,255	971	372	763,100	539,790	92.53	37.48
D_1524A	1,524	D	Rumen fluid	210,080,000	200,600,400	200,293,646	306,754	0.15	623,258	502,312,670	931	369	871,981	604,834	92.39	38.01
D_2297A	2,297	D	Rumen fluid	209,600,000	200,531,708	199,325,160	1,206,548	0.60	619,764	470,176,627	865	362	802,084	552,985	92.44	35.81
D_2325A	2,325	D	Rumen fluid	211,760,000	200,614,322	200,468,118	146,204	0.07	638,393	494,277,606	888	365	805,639	533,967	92.45	33.69
D_2331A	2,331	D	Rumen fluid	211,520,000	200,603,726	199,678,498	925,228	0.46	651,398	495,311,701	865	367	828,255	574,368	92.45	36.09
D_2357A	2,357	D	Rumen fluid	212,480,000	200,665,468	200,452,378	213,090	0.11	600,059	454,295,917	878	360	768,766	503,200	92.51	34.48
D_9633A	9,633	D	Rumen fluid	212,800,000	200,592,604	199,045,304	1,547,300	0.77	603,220	472,764,978	896	372	787,320	541,458	92.40	37.56
E_1506A	1,506	E	Rumen fluid	212,560,000	200,457,240	199,830,514	626,726	0.31	571,318	456,168,532	908	369	772,924	547,273	92.14	37.74
E_2307A	2,307	E	Rumen fluid	210,960,000	200,782,228	200,448,920	333,308	0.17	623,796	452,309,033	812	361	784,536	538,848	92.85	35.87
E_2327A	2,327	E	Rumen fluid	214,000,000	200,623,158	200,202,712	420,446	0.21	548,837	415,890,373	850	363	730,502	515,093	92.50	39.03
E_2329A	2,329	E	Rumen fluid	213,600,000	200,623,350	200,338,794	284,556	0.14	556,804	442,713,697	880	375	747,013	550,997	92.42	39.70
E_2375A	2,375	E	Rumen fluid	213,360,000	200,653,364	200,506,816	146,548	0.07	666,031	530,007,533	899	368	925,028	696,335	92.38	39.28
E_9611A	9,611	E	Rumen fluid	214,080,000	200,596,326	200,303,872	292,454	0.15	584,870	466,318,772	907	367	808,291	587,820	92.31	39.03
CE_A2315A	2,315	A	Cecum	233,440,000	200,428,060	171,170,278	29,257,782	14.60	605,035	529,945,850	943	425	971,365	846,772	93.39	45.93
CE_A9613A	9,613	A	Cecum	224,320,000	200,400,450	176,879,404	23,521,046	11.74	620,915	579,552,327	1,055	438	1,029,643	895,758	93.13	45.89
CE_C1512A	1,512	C	Cecum	223,680,000	200,507,358	100,642,708	99,864,650	49.81	305,499	301,517,014	1,231	414	507,944	406,269	93.16	45.64
CE_2339A	2,339	C	Cecum	222,720,000	200,442,432	150,367,094	50,075,338	24.98	414,517	474,139,517	1,566	495	755,306	622,157	90.63	44.23
CE_C2349A	2,349	C	Cecum	215,760,000	200,634,520	139,835,212	60,799,308	30.30	447,340	449,339,062	1,230	435	764,296	655,480	92.60	45.24
CO_A2315B	2,315	A	Colon	288,800,000	200,288,546	77,060,788	123,227,758	61.53	190,041	157,944,215	928	387	320,491	279,279	92.69	56.48
CO_A9613A	9,613	A	Colon	221,120,000	200,562,444	126,803,428	73,759,016	36.78	371,762	348,317,169	1,064	415	627,123	535,917	92.71	45.05
CO_C1512A	1,512	C	Colon	218,000,000	200,539,034	173,995,300	26,543,734	13.24	624,173	618,452,604	1,258	412	1,050,357	872,513	92.97	46.55
CO_2339A	2,339	C	Colon	212,800,000	200,424,758	178,947,024	21,477,734	10.72	459,581	554,247,643	1,782	495	859,107	707,986	90.73	44.96
CO_C2349A	2,349	C	Colon	220,640,000	200,445,006	99,212,878	101,232,128	50.50	299,435	288,673,865	1,095	456	501,199	422,079	93.69	45.40
DM_A2315A	2,315	A	Duodenum	232,400,000	200,531,704	3,778,348	196,753,356	98.12	9,950	8,614,804	989	443	9,197	8,387	92.95	44.74
DM_A9613A	9,613	A	Duodenum	216,080,000	200,674,596	2,634,284	198,040,312	98.69	10,149	8,744,633	974	454	9,147	8,333	92.89	44.58
DM_2339A	2,339	C	Duodenum	207,680,000	200,624,182	2,798,010	197,826,172	98.61	9,680	8,459,198	988	453	9,266	8,415	91.05	43.61
IM_A2315A	2,315	A	Ileum	211,920,000	200,599,028	3,149,652	197,449,376	98.43	9,619	8,983,975	1,099	471	9,318	8,499	92.57	43.85
IM_A9613A	9,613	A	Ileum	206,880,000	200,581,082	4,897,370	195,683,712	97.56	10,191	9,016,701	1,018	472	9,476	8,656	92.61	43.66
JM_A2315A	2,315	A	Jejunum	212,400,000	200,753,616	2,863,352	197,890,264	98.57	9,897	9,059,648	1,088	472	9,486	8,647	92.32	43.37
JM_A9613A	9,613	A	Jejunum	207,600,000	200,465,248	2,546,442	197,918,806	98.73	9,359	8,828,857	1,109	478	9,164	8,363	91.94	43.52
JM_C1512A	1,512	C	Jejunum	210,960,000	200,651,012	2,807,230	197,843,782	98.60	9,738	9,344,839	1,165	472	9,839	8,961	92.27	43.39
JM_C2349A	2,349	C	Jejunum	207,520,000	200,774,556	2,977,042	197,797,514	98.52	9,395	9,010,337	1,136	487	9,229	8,375	92.07	43.52
R_A2315A	2,315	A	Rumen content	212,640,000	200,501,750	200,299,444	202,306	0.10	561,280	438,974,234	852	365	831,318	659,245	92.20	44.82
R_A9613A	9,613	A	Rumen content	212,160,000	200,649,988	200,356,958	293,030	0.15	605,344	502,327,141	974	370	873,971	655,747	92.43	40.57
R_C1512A	1,512	C	Rumen content	208,160,000	200,554,234	200,204,124	350,110	0.17	545,372	423,155,295	840	361	811,478	636,429	92.65	45.83
R_2339A	2,339	C	Rumen content	206,400,000	200,439,200	200,248,210	190,990	0.10	553,741	451,374,516	924	372	779,125	581,163	90.74	41.21
R_C2349A	2,349	C	Rumen content	208,160,000	200,772,716	199,899,106	873,610	0.44	693,983	600,910,592	1,029	371	1,031,885	805,886	91.99	43.04

https://doi.org/10.3390/microorganisms14102206

Note: Read counts are individual reads, not read pairs. Post-host reads are reads retained after host alignment filtering. Host-read counts and percentages are calculated from clean and post-host counts. Contig lengths and N50/N90 are in base pairs. The CDS and redundancy-filtered CDS columns are sample-level counts and should not be summed to obtain the size of a pooled non-redundant catalog.

Table S5. Rumen-only genus–function correlations

genus	functional_feature	n	Spearman_rho	permutation_P	BH_q	q_lt_0_05	permutations
Xanthomonas	KEGG:Carbohydrate_metabolism	30	-0.7949	0.001000	0.002113	*	999
Xanthomonas	KEGG:Amino_acid_metabolism	30	-0.7646	0.001000	0.002113	*	999
Xanthomonas	KEGG:Energy_metabolism	30	-0.8229	0.001000	0.002113	*	999
Xanthomonas	KEGG:Glycan_biosynthesis_and_metabolism	30	-0.6494	0.001000	0.002113	*	999
Xanthomonas	KEGG:Lipid_metabolism	30	-0.7477	0.001000	0.002113	*	999
Xanthomonas	KEGG:Metabolism_of_cofactors_and_vitamins	30	-0.8038	0.001000	0.002113	*	999
Xanthomonas	KEGG:Membrane_transport	30	-0.8732	0.001000	0.002113	*	999
Xanthomonas	CAZy:CBM37	30	0.8870	0.001000	0.002113	*	999
Xanthomonas	CAZy:GT2	30	-0.7424	0.001000	0.002113	*	999
Xanthomonas	CAZy:GT4	30	-0.5417	0.003000	0.005556	*	999
Xanthomonas	CAZy:GH13	30	0.0078	0.977000	0.977000		999
Xanthomonas	CAZy:CBM50	30	-0.7264	0.001000	0.002113	*	999
Xanthomonas	CAZy:GH16	30	0.7882	0.001000	0.002113	*	999
Xanthomonas	CAZy:GH43	30	-0.8514	0.001000	0.002113	*	999
Xanthomonas	CAZy:GT9	30	-0.8047	0.001000	0.002113	*	999
Prevotella	KEGG:Carbohydrate_metabolism	30	0.8363	0.001000	0.002113	*	999
Prevotella	KEGG:Amino_acid_metabolism	30	0.8118	0.001000	0.002113	*	999
Prevotella	KEGG:Energy_metabolism	30	0.7913	0.001000	0.002113	*	999
Prevotella	KEGG:Glycan_biosynthesis_and_metabolism	30	0.8105	0.001000	0.002113	*	999
Prevotella	KEGG:Lipid_metabolism	30	0.8256	0.001000	0.002113	*	999
Prevotella	KEGG:Metabolism_of_cofactors_and_vitamins	30	0.8487	0.001000	0.002113	*	999
Prevotella	KEGG:Membrane_transport	30	0.7695	0.001000	0.002113	*	999
Prevotella	CAZy:CBM37	30	-0.6276	0.001000	0.002113	*	999
Prevotella	CAZy:GT2	30	0.8616	0.001000	0.002113	*	999
Prevotella	CAZy:GT4	30	0.7482	0.001000	0.002113	*	999
Prevotella	CAZy:GH13	30	0.3602	0.052000	0.067826		999
Prevotella	CAZy:CBM50	30	0.8225	0.001000	0.002113	*	999
Prevotella	CAZy:GH16	30	-0.6258	0.002000	0.003822	*	999
Prevotella	CAZy:GH43	30	0.7597	0.001000	0.002113	*	999
Prevotella	CAZy:GT9	30	0.7806	0.001000	0.002113	*	999
Xylanibacter	KEGG:Carbohydrate_metabolism	30	0.6703	0.001000	0.002113	*	999
Xylanibacter	KEGG:Amino_acid_metabolism	30	0.6320	0.001000	0.002113	*	999
Xylanibacter	KEGG:Energy_metabolism	30	0.6774	0.001000	0.002113	*	999
Xylanibacter	KEGG:Glycan_biosynthesis_and_metabolism	30	0.5368	0.001000	0.002113	*	999
Xylanibacter	KEGG:Lipid_metabolism	30	0.6254	0.001000	0.002113	*	999

genus	functional_feature	n	Spearman_rho	permutation_P	BH_q	q_lt_0_05	permutations
Xylanibacter	KEGG:Metabolism_of_cofactors_and_vitamins	30	0.6565	0.002000	0.003822	*	999
Xylanibacter	KEGG:Membrane_transport	30	0.7059	0.001000	0.002113	*	999
Xylanibacter	CAZy:CBM37	30	-0.6525	0.001000	0.002113	*	999
Xylanibacter	CAZy:GT2	30	0.6392	0.001000	0.002113	*	999
Xylanibacter	CAZy:GT4	30	0.4612	0.015000	0.023077	*	999
Xylanibacter	CAZy:GH13	30	0.1204	0.525000	0.568592		999
Xylanibacter	CAZy:CBM50	30	0.6303	0.001000	0.002113	*	999
Xylanibacter	CAZy:GH16	30	-0.5488	0.006000	0.010227	*	999
Xylanibacter	CAZy:GH43	30	0.8100	0.001000	0.002113	*	999
Xylanibacter	CAZy:GT9	30	0.6044	0.002000	0.003822	*	999
Fibrobacter	KEGG:Carbohydrate_metabolism	30	0.2703	0.138000	0.164940		999
Fibrobacter	KEGG:Amino_acid_metabolism	30	0.2908	0.119000	0.143952		999
Fibrobacter	KEGG:Energy_metabolism	30	0.3406	0.069000	0.088085		999
Fibrobacter	KEGG:Glycan_biosynthesis_and_metabolism	30	0.1657	0.360000	0.401487		999
Fibrobacter	KEGG:Lipid_metabolism	30	0.2765	0.134000	0.160800		999
Fibrobacter	KEGG:Metabolism_of_cofactors_and_vitamins	30	0.3281	0.091000	0.112346		999
Fibrobacter	KEGG:Membrane_transport	30	0.4834	0.011000	0.017460	*	999
Fibrobacter	CAZy:CBM37	30	-0.5346	0.003000	0.005556	*	999
Fibrobacter	CAZy:GT2	30	0.2712	0.140000	0.166008		999
Fibrobacter	CAZy:GT4	30	0.0345	0.856000	0.876451		999
Fibrobacter	CAZy:GH13	30	-0.3353	0.082000	0.102500		999
Fibrobacter	CAZy:CBM50	30	0.1849	0.307000	0.351527		999
Fibrobacter	CAZy:GH16	30	-0.5199	0.002000	0.003822	*	999
Fibrobacter	CAZy:GH43	30	0.4603	0.013000	0.020419	*	999
Fibrobacter	CAZy:GT9	30	0.3726	0.039000	0.052703		999
Bacillus	KEGG:Carbohydrate_metabolism	30	-0.4349	0.021000	0.030000	*	999
Bacillus	KEGG:Amino_acid_metabolism	30	-0.4358	0.016000	0.024242	*	999
Bacillus	KEGG:Energy_metabolism	30	-0.4505	0.009000	0.014595	*	999
Bacillus	KEGG:Glycan_biosynthesis_and_metabolism	30	-0.3766	0.037000	0.050226		999
Bacillus	KEGG:Lipid_metabolism	30	-0.4180	0.018000	0.026471	*	999
Bacillus	KEGG:Metabolism_of_cofactors_and_vitamins	30	-0.4710	0.009000	0.014595	*	999
Bacillus	KEGG:Membrane_transport	30	-0.5782	0.001000	0.002113	*	999
Bacillus	CAZy:CBM37	30	0.5982	0.001000	0.002113	*	999
Bacillus	CAZy:GT2	30	-0.4839	0.011000	0.017460	*	999
Bacillus	CAZy:GT4	30	-0.3157	0.091000	0.112346		999
Bacillus	CAZy:GH13	30	0.1315	0.472000	0.514909		999
Bacillus	CAZy:CBM50	30	-0.4741	0.012000	0.018947	*	999

genus	functional_feature	n	Spearman_rho	permutation_P	BH_q	q_lt_0_05	permutations
Bacillus	CAZy:GH16	30	0.5973	0.002000	0.003822	*	999
Bacillus	CAZy:GH43	30	-0.6663	0.001000	0.002113	*	999
Bacillus	CAZy:GT9	30	-0.4220	0.021000	0.030000	*	999
Staphylococcus	KEGG:Carbohydrate_metabolism	30	-0.7477	0.001000	0.002113	*	999
Staphylococcus	KEGG:Amino_acid_metabolism	30	-0.7059	0.001000	0.002113	*	999
Staphylococcus	KEGG:Energy_metabolism	30	-0.6974	0.001000	0.002113	*	999
Staphylococcus	KEGG:Glycan_biosynthesis_and_metabolism	30	-0.7126	0.001000	0.002113	*	999
Staphylococcus	KEGG:Lipid_metabolism	30	-0.7086	0.001000	0.002113	*	999
Staphylococcus	KEGG:Metabolism_of_cofactors_and_vitamins	30	-0.6939	0.001000	0.002113	*	999
Staphylococcus	KEGG:Membrane_transport	30	-0.5951	0.001000	0.002113	*	999
Staphylococcus	CAZy:CBM37	30	0.5462	0.002000	0.003822	*	999
Staphylococcus	CAZy:GT2	30	-0.6699	0.002000	0.003822	*	999
Staphylococcus	CAZy:GT4	30	-0.6747	0.001000	0.002113	*	999
Staphylococcus	CAZy:GH13	30	-0.4438	0.009000	0.014595	*	999
Staphylococcus	CAZy:CBM50	30	-0.7749	0.001000	0.002113	*	999
Staphylococcus	CAZy:GH16	30	0.4029	0.037000	0.050226		999
Staphylococcus	CAZy:GH43	30	-0.6836	0.001000	0.002113	*	999
Staphylococcus	CAZy:GT9	30	-0.6276	0.003000	0.005556	*	999
Bacteroides	KEGG:Carbohydrate_metabolism	30	0.8532	0.001000	0.002113	*	999
Bacteroides	KEGG:Amino_acid_metabolism	30	0.8594	0.001000	0.002113	*	999
Bacteroides	KEGG:Energy_metabolism	30	0.8171	0.001000	0.002113	*	999
Bacteroides	KEGG:Glycan_biosynthesis_and_metabolism	30	0.8937	0.001000	0.002113	*	999
Bacteroides	KEGG:Lipid_metabolism	30	0.8723	0.001000	0.002113	*	999
Bacteroides	KEGG:Metabolism_of_cofactors_and_vitamins	30	0.8509	0.001000	0.002113	*	999
Bacteroides	KEGG:Membrane_transport	30	0.7651	0.001000	0.002113	*	999
Bacteroides	CAZy:CBM37	30	-0.6788	0.001000	0.002113	*	999
Bacteroides	CAZy:GT2	30	0.8274	0.001000	0.002113	*	999
Bacteroides	CAZy:GT4	30	0.8412	0.001000	0.002113	*	999
Bacteroides	CAZy:GH13	30	0.4278	0.034000	0.047005	*	999
Bacteroides	CAZy:CBM50	30	0.8229	0.001000	0.002113	*	999
Bacteroides	CAZy:GH16	30	-0.6000	0.001000	0.002113	*	999
Bacteroides	CAZy:GH43	30	0.6334	0.002000	0.003822	*	999
Bacteroides	CAZy:GT9	30	0.7900	0.001000	0.002113	*	999
Clostridium	KEGG:Carbohydrate_metabolism	30	-0.8225	0.001000	0.002113	*	999
Clostridium	KEGG:Amino_acid_metabolism	30	-0.8091	0.001000	0.002113	*	999
Clostridium	KEGG:Energy_metabolism	30	-0.8305	0.001000	0.002113	*	999
Clostridium	KEGG:Glycan_biosynthesis_and_metabolism	30	-0.7611	0.001000	0.002113	*	999
Clostridium	KEGG:Lipid_metabolism	30	-0.8189	0.001000	0.002113	*	999
Clostridium	KEGG:Metabolism_of_cofactors_and_vitamins	30	-0.8469	0.001000	0.002113	*	999
Clostridium	KEGG:Membrane_transport	30	-0.8892	0.001000	0.002113	*	999
Clostridium	CAZy:CBM37	30	0.7744	0.001000	0.002113	*	999
Clostridium	CAZy:GT2	30	-0.8287	0.001000	0.002113	*	999
Clostridium	CAZy:GT4	30	-0.6659	0.001000	0.002113	*	999

genus	functional_feature	n	Spearman_rho	permutation_P	BH_q	q_lt_0_05	permuta-tions
Clostridium	CAZy:GH13	30	-0.2085	0.262000	0.302308		999
Clostridium	CAZy:CBM50	30	-0.7517	0.001000	0.002113	*	999
Clostridium	CAZy:GH16	30	0.7967	0.001000	0.002113	*	999
Clostridium	CAZy:GH43	30	-0.8590	0.001000	0.002113	*	999
Clostridium	CAZy:GT9	30	-0.8242	0.001000	0.002113	*	999
Segatella	KEGG:Carbohydrate_metabolism	30	0.6899	0.001000	0.002113	*	999
Segatella	KEGG:Amino_acid_metabolism	30	0.6650	0.001000	0.002113	*	999
Segatella	KEGG:Energy_metabolism	30	0.6512	0.001000	0.002113	*	999
Segatella	KEGG:Glycan_biosynthesis_and_metabolism	30	0.6943	0.001000	0.002113	*	999
Segatella	KEGG:Lipid_metabolism	30	0.6877	0.001000	0.002113	*	999
Segatella	KEGG:Metabolism_of_cofactors_and_vitamins	30	0.7112	0.001000	0.002113	*	999
Segatella	KEGG:Membrane_transport	30	0.6356	0.002000	0.003822	*	999
Segatella	CAZy:CBM37	30	-0.5471	0.003000	0.005556	*	999
Segatella	CAZy:GT2	30	0.7624	0.001000	0.002113	*	999
Segatella	CAZy:GT4	30	0.6645	0.001000	0.002113	*	999
Segatella	CAZy:GH13	30	0.1729	0.329000	0.371053		999
Segatella	CAZy:CBM50	30	0.6672	0.001000	0.002113	*	999
Segatella	CAZy:GH16	30	-0.5737	0.001000	0.002113	*	999
Segatella	CAZy:GH43	30	0.5947	0.003000	0.005556	*	999
Segatella	CAZy:GT9	30	0.6476	0.001000	0.002113	*	999
Escherichia	KEGG:Carbohydrate_metabolism	30	-0.3922	0.030000	0.041667	*	999
Escherichia	KEGG:Amino_acid_metabolism	30	-0.3815	0.045000	0.060268		999
Escherichia	KEGG:Energy_metabolism	30	-0.4091	0.025000	0.035211	*	999
Escherichia	KEGG:Glycan_biosynthesis_and_metabolism	30	-0.4087	0.025000	0.035211	*	999
Escherichia	KEGG:Lipid_metabolism	30	-0.3984	0.035000	0.048165	*	999
Escherichia	KEGG:Metabolism_of_cofactors_and_vitamins	30	-0.4420	0.019000	0.027805	*	999
Escherichia	KEGG:Membrane_transport	30	-0.4394	0.015000	0.023077	*	999
Escherichia	CAZy:CBM37	30	0.4358	0.021000	0.030000	*	999
Escherichia	CAZy:GT2	30	-0.4581	0.017000	0.025373	*	999
Escherichia	CAZy:GT4	30	-0.3135	0.094000	0.115102		999
Escherichia	CAZy:GH13	30	-0.1017	0.589000	0.624382		999
Escherichia	CAZy:CBM50	30	-0.3348	0.074000	0.094068		999
Escherichia	CAZy:GH16	30	0.4398	0.017000	0.025373	*	999
Escherichia	CAZy:GH43	30	-0.3958	0.040000	0.053812		999
Escherichia	CAZy:GT9	30	-0.3993	0.027000	0.037850	*	999
Aristaeella	KEGG:Carbohydrate_metabolism	30	0.3535	0.051000	0.066812		999
Aristaeella	KEGG:Amino_acid_metabolism	30	0.3326	0.077000	0.096653		999
Aristaeella	KEGG:Energy_metabolism	30	0.4274	0.018000	0.026471	*	999
Aristaeella	KEGG:Glycan_biosynthesis_and_metabolism	30	0.2676	0.151000	0.178346		999

genus	functional_feature	n	Spearman_rho	permutation_P	BH_q	q_lt_0_05	permutations
Aristaeella	KEGG:Lipid_metabolism	30	0.3295	0.076000	0.095798		999
Aristaeella	KEGG:Metabolism_of_cofactors_and_vitamins	30	0.3419	0.076000	0.095798		999
Aristaeella	KEGG:Membrane_transport	30	0.5373	0.005000	0.008621	*	999
Aristaeella	CAZy:CBM37	30	-0.5475	0.006000	0.010227	*	999
Aristaeella	CAZy:GT2	30	0.3682	0.048000	0.063158		999
Aristaeella	CAZy:GT4	30	0.2191	0.262000	0.302308		999
Aristaeella	CAZy:GH13	30	-0.2556	0.169000	0.198047		999
Aristaeella	CAZy:CBM50	30	0.2863	0.139000	0.165476		999
Aristaeella	CAZy:GH16	30	-0.5101	0.005000	0.008621	*	999
Aristaeella	CAZy:GH43	30	0.5978	0.001000	0.002113	*	999
Aristaeella	CAZy:GT9	30	0.4309	0.022000	0.031280	*	999
Plasmodium	KEGG:Carbohydrate_metabolism	30	-0.8888	0.001000	0.002113	*	999
Plasmodium	KEGG:Amino_acid_metabolism	30	-0.8834	0.001000	0.002113	*	999
Plasmodium	KEGG:Energy_metabolism	30	-0.9132	0.001000	0.002113	*	999
Plasmodium	KEGG:Glycan_biosynthesis_and_metabolism	30	-0.8158	0.001000	0.002113	*	999
Plasmodium	KEGG:Lipid_metabolism	30	-0.8794	0.001000	0.002113	*	999
Plasmodium	KEGG:Metabolism_of_cofactors_and_vitamins	30	-0.9208	0.001000	0.002113	*	999
Plasmodium	KEGG:Membrane_transport	30	-0.9488	0.001000	0.002113	*	999
Plasmodium	CAZy:CBM37	30	0.8545	0.001000	0.002113	*	999
Plasmodium	CAZy:GT2	30	-0.8652	0.001000	0.002113	*	999
Plasmodium	CAZy:GT4	30	-0.6863	0.001000	0.002113	*	999
Plasmodium	CAZy:GH13	30	-0.1693	0.358000	0.401487		999
Plasmodium	CAZy:CBM50	30	-0.7984	0.001000	0.002113	*	999
Plasmodium	CAZy:GH16	30	0.8705	0.001000	0.002113	*	999
Plasmodium	CAZy:GH43	30	-0.8754	0.001000	0.002113	*	999
Plasmodium	CAZy:GT9	30	-0.9275	0.001000	0.002113	*	999
Nostoc	KEGG:Carbohydrate_metabolism	30	-0.7927	0.001000	0.002113	*	999
Nostoc	KEGG:Amino_acid_metabolism	30	-0.7660	0.001000	0.002113	*	999
Nostoc	KEGG:Energy_metabolism	30	-0.8056	0.001000	0.002113	*	999
Nostoc	KEGG:Glycan_biosynthesis_and_metabolism	30	-0.7072	0.001000	0.002113	*	999
Nostoc	KEGG:Lipid_metabolism	30	-0.7660	0.001000	0.002113	*	999
Nostoc	KEGG:Metabolism_of_cofactors_and_vitamins	30	-0.7998	0.001000	0.002113	*	999
Nostoc	KEGG:Membrane_transport	30	-0.8661	0.001000	0.002113	*	999
Nostoc	CAZy:CBM37	30	0.8732	0.001000	0.002113	*	999
Nostoc	CAZy:GT2	30	-0.7953	0.001000	0.002113	*	999
Nostoc	CAZy:GT4	30	-0.6102	0.002000	0.003822	*	999
Nostoc	CAZy:GH13	30	-0.0171	0.907000	0.925510		999
Nostoc	CAZy:CBM50	30	-0.7517	0.001000	0.002113	*	999
Nostoc	CAZy:GH16	30	0.8069	0.001000	0.002113	*	999
Nostoc	CAZy:GH43	30	-0.8603	0.001000	0.002113	*	999

https://doi.org/10.3390/microorganisms14102206

genus	functional_feature	n	Spearman_rho	permutation_P	BH_q	q_lt_0_05	permuta-tions
Nostoc	CAZy:GT9	30	-0.7802	0.001000	0.002113	*	999
Alistipes	KEGG:Carbohydrate_metabolism	30	0.8314	0.001000	0.002113	*	999
Alistipes	KEGG:Amino_acid_metabolism	30	0.8229	0.001000	0.002113	*	999
Alistipes	KEGG:Energy_metabolism	30	0.8505	0.001000	0.002113	*	999
Alistipes	KEGG:Glycan_biosynthesis_and_metabolism	30	0.8412	0.001000	0.002113	*	999
Alistipes	KEGG:Lipid_metabolism	30	0.8434	0.001000	0.002113	*	999
Alistipes	KEGG:Metabolism_of_cofactors_and_vitamins	30	0.8331	0.001000	0.002113	*	999
Alistipes	KEGG:Membrane_transport	30	0.8047	0.001000	0.002113	*	999
Alistipes	CAZy:CBM37	30	-0.8078	0.001000	0.002113	*	999
Alistipes	CAZy:GT2	30	0.8598	0.001000	0.002113	*	999
Alistipes	CAZy:GT4	30	0.7793	0.001000	0.002113	*	999
Alistipes	CAZy:GH13	30	0.1929	0.309000	0.352471		999
Alistipes	CAZy:CBM50	30	0.8354	0.001000	0.002113	*	999
Alistipes	CAZy:GH16	30	-0.7081	0.001000	0.002113	*	999
Alistipes	CAZy:GH43	30	0.7762	0.001000	0.002113	*	999
Alistipes	CAZy:GT9	30	0.8260	0.001000	0.002113	*	999
Streptococcus	KEGG:Carbohydrate_metabolism	30	-0.4945	0.005000	0.008621	*	999
Streptococcus	KEGG:Amino_acid_metabolism	30	-0.5350	0.004000	0.007143	*	999
Streptococcus	KEGG:Energy_metabolism	30	-0.5119	0.004000	0.007143	*	999
Streptococcus	KEGG:Glycan_biosynthesis_and_metabolism	30	-0.5052	0.004000	0.007143	*	999
Streptococcus	KEGG:Lipid_metabolism	30	-0.5328	0.004000	0.007143	*	999
Streptococcus	KEGG:Metabolism_of_cofactors_and_vitamins	30	-0.5128	0.002000	0.003822	*	999
Streptococcus	KEGG:Membrane_transport	30	-0.5208	0.005000	0.008621	*	999
Streptococcus	CAZy:CBM37	30	0.4474	0.017000	0.025373	*	999
Streptococcus	CAZy:GT2	30	-0.5359	0.002000	0.003822	*	999
Streptococcus	CAZy:GT4	30	-0.4554	0.015000	0.023077	*	999
Streptococcus	CAZy:GH13	30	-0.0607	0.742000	0.772917		999
Streptococcus	CAZy:CBM50	30	-0.5110	0.005000	0.008621	*	999
Streptococcus	CAZy:GH16	30	0.4225	0.021000	0.030000	*	999
Streptococcus	CAZy:GH43	30	-0.5119	0.005000	0.008621	*	999
Streptococcus	CAZy:GT9	30	-0.4790	0.007000	0.011798	*	999
Klebsiella	KEGG:Carbohydrate_metabolism	30	-0.3762	0.037000	0.050226		999
Klebsiella	KEGG:Amino_acid_metabolism	30	-0.3584	0.055000	0.071121		999
Klebsiella	KEGG:Energy_metabolism	30	-0.3669	0.055000	0.071121		999
Klebsiella	KEGG:Glycan_biosynthesis_and_metabolism	30	-0.3095	0.094000	0.115102		999
Klebsiella	KEGG:Lipid_metabolism	30	-0.3415	0.059000	0.075641		999
Klebsiella	KEGG:Metabolism_of_cofactors_and_vitamins	30	-0.3878	0.029000	0.040465	*	999
Klebsiella	KEGG:Membrane_transport	30	-0.4541	0.016000	0.024242	*	999
Klebsiella	CAZy:CBM37	30	0.5106	0.004000	0.007143	*	999
Klebsiella	CAZy:GT2	30	-0.4251	0.016000	0.024242	*	999

https://doi.org/10.3390/microorganisms14102206

genus	functional_feature	n	Spearman_rho	permutation_P	BH_q	q_lt_0_05	permuta-tions
Klebsiella	CAZy:GT4	30	-0.2369	0.184000	0.214786		999
Klebsiella	CAZy:GH13	30	0.0768	0.673000	0.705944		999
Klebsiella	CAZy:CBM50	30	-0.4247	0.018000	0.026471	*	999
Klebsiella	CAZy:GH16	30	0.4483	0.007000	0.011798	*	999
Klebsiella	CAZy:GH43	30	-0.4905	0.009000	0.014595	*	999
Klebsiella	CAZy:GT9	30	-0.3686	0.046000	0.061333		999
Streptomyces	KEGG:Carbohydrate_metabolism	30	0.7188	0.001000	0.002113	*	999
Streptomyces	KEGG:Amino_acid_metabolism	30	0.7339	0.001000	0.002113	*	999
Streptomyces	KEGG:Energy_metabolism	30	0.7268	0.001000	0.002113	*	999
Streptomyces	KEGG:Glycan_biosynthesis_and_metabolism	30	0.6734	0.001000	0.002113	*	999
Streptomyces	KEGG:Lipid_metabolism	30	0.7299	0.001000	0.002113	*	999
Streptomyces	KEGG:Metabolism_of_cofactors_and_vitamins	30	0.6912	0.001000	0.002113	*	999
Streptomyces	KEGG:Membrane_transport	30	0.7259	0.001000	0.002113	*	999
Streptomyces	CAZy:CBM37	30	-0.7798	0.001000	0.002113	*	999
Streptomyces	CAZy:GT2	30	0.6805	0.002000	0.003822	*	999
Streptomyces	CAZy:GT4	30	0.6160	0.001000	0.002113	*	999
Streptomyces	CAZy:GH13	30	0.1947	0.303000	0.348276		999
Streptomyces	CAZy:CBM50	30	0.6948	0.001000	0.002113	*	999
Streptomyces	CAZy:GH16	30	-0.6409	0.002000	0.003822	*	999
Streptomyces	CAZy:GH43	30	0.6289	0.002000	0.003822	*	999
Streptomyces	CAZy:GT9	30	0.7197	0.001000	0.002113	*	999
Salmonella	KEGG:Carbohydrate_metabolism	30	-0.1404	0.440000	0.485294		999
Salmonella	KEGG:Amino_acid_metabolism	30	-0.0945	0.613000	0.645263		999
Salmonella	KEGG:Energy_metabolism	30	-0.1008	0.570000	0.608541		999
Salmonella	KEGG:Glycan_biosynthesis_and_metabolism	30	-0.1729	0.360000	0.401487		999
Salmonella	KEGG:Lipid_metabolism	30	-0.1066	0.550000	0.591398		999
Salmonella	KEGG:Metabolism_of_cofactors_and_vitamins	30	-0.1088	0.597000	0.630634		999
Salmonella	KEGG:Membrane_transport	30	-0.1119	0.542000	0.584892		999
Salmonella	CAZy:CBM37	30	0.1555	0.425000	0.470480		999
Salmonella	CAZy:GT2	30	-0.1404	0.458000	0.503297		999
Salmonella	CAZy:GT4	30	-0.2445	0.227000	0.263953		999
Salmonella	CAZy:GH13	30	-0.1359	0.481000	0.522826		999
Salmonella	CAZy:CBM50	30	-0.1875	0.318000	0.361364		999
Salmonella	CAZy:GH16	30	0.1511	0.407000	0.452222		999
Salmonella	CAZy:GH43	30	-0.0781	0.692000	0.723345		999

https://doi.org/10.3390/microorganisms14102206

genus	functional_feature	n	Spearman_rho	permutation_P	BH_q	q_lt_0_05	permutations
Salmonella	CAZy:GT9	30	-0.1404	0.463000	0.506934		999
Butyrivibrio	KEGG:Carbohydrate_metabolism	30	0.4612	0.014000	0.021875	*	999
Butyrivibrio	KEGG:Amino_acid_metabolism	30	0.4892	0.010000	0.016129	*	999
Butyrivibrio	KEGG:Energy_metabolism	30	0.5190	0.004000	0.007143	*	999
Butyrivibrio	KEGG:Glycan_biosynthesis_and_metabolism	30	0.3544	0.057000	0.073391		999
Butyrivibrio	KEGG:Lipid_metabolism	30	0.4501	0.011000	0.017460	*	999
Butyrivibrio	KEGG:Metabolism_of_cofactors_and_vitamins	30	0.4545	0.009000	0.014595	*	999
Butyrivibrio	KEGG:Membrane_transport	30	0.5809	0.001000	0.002113	*	999
Butyrivibrio	CAZy:CBM37	30	-0.3606	0.047000	0.062389		999
Butyrivibrio	CAZy:GT2	30	0.4469	0.020000	0.029126	*	999
Butyrivibrio	CAZy:GT4	30	0.3246	0.084000	0.104564		999
Butyrivibrio	CAZy:GH13	30	-0.0634	0.752000	0.780623		999
Butyrivibrio	CAZy:CBM50	30	0.3050	0.108000	0.131174		999
Butyrivibrio	CAZy:GH16	30	-0.4679	0.009000	0.014595	*	999
Butyrivibrio	CAZy:GH43	30	0.6031	0.001000	0.002113	*	999
Butyrivibrio	CAZy:GT9	30	0.4768	0.008000	0.013408	*	999
Enterococcus	KEGG:Carbohydrate_metabolism	30	-0.0078	0.968000	0.971237		999
Enterococcus	KEGG:Amino_acid_metabolism	30	0.0122	0.947000	0.956566		999
Enterococcus	KEGG:Energy_metabolism	30	-0.0247	0.915000	0.930508		999
Enterococcus	KEGG:Glycan_biosynthesis_and_metabolism	30	0.0558	0.762000	0.788276		999
Enterococcus	KEGG:Lipid_metabolism	30	0.0167	0.932000	0.944595		999
Enterococcus	KEGG:Metabolism_of_cofactors_and_vitamins	30	-0.0585	0.767000	0.790722		999
Enterococcus	KEGG:Membrane_transport	30	-0.1924	0.321000	0.363396		999
Enterococcus	CAZy:CBM37	30	0.2983	0.101000	0.123171		999
Enterococcus	CAZy:GT2	30	-0.1017	0.574000	0.610638		999
Enterococcus	CAZy:GT4	30	0.1115	0.557000	0.596786		999
Enterococcus	CAZy:GH13	30	0.3055	0.123000	0.148193		999
Enterococcus	CAZy:CBM50	30	-0.0091	0.961000	0.967450		999
Enterococcus	CAZy:GH16	30	0.3784	0.048000	0.063158		999
Enterococcus	CAZy:GH43	30	-0.2538	0.167000	0.196471		999
Enterococcus	CAZy:GT9	30	-0.0398	0.826000	0.848630		999

Note: The 300 rows provide Spearman coefficients, permutation P values, and Benjamini–Hochberg q values for 20 genera and 15 functional features across 30 rumen-fluid samples. The 15 features comprise seven KEGG Level 2 categories and eight CAZyme families. The asterisk column marks q<0.05; the matrix contains 218 such pairs.

https://doi.org/10.3390/microorganisms14102206
